# Synergistic Interactions in a Heterobimetallic Ce(III)–Ni(II) Diimine Complex: Enhancing the Electrocatalytic Efficiency for CO_2_ Reduction

**DOI:** 10.1021/acsaem.4c02132

**Published:** 2024-10-21

**Authors:** Farzaneh Yari, Abdalaziz Aljabour, Houssein Awada, Jessica Michalke, Nidhi Kumari, Halime Coskun-Aljabour, Soumyajit Roy, Dominik Krisch, Wolfgang Schöfberger

**Affiliations:** † Institute of Organic Chemistry, Laboratory for Sustainable Chemistry and Catalysis (LSusCat), Johannes Kepler University (JKU), Altenberger Straße 69, 4040 Linz, Austria; ‡ Chair of Physical Chemistry, Montanuniversität Leoben, 8700 Leoben, Austria; § Institute for Catalysis (INCA), Johannes Kepler University, 4040 Linz, Austria; ∥ Eco-Friendly Applied Materials Laboratory, Department of Chemical Sciences, Materials Science Centre, Indian Institute of Science Education and Research, Kolkata 741246, West Bengal, India

**Keywords:** CO_2_ electrocatalysis, salen ligand, cerium nickel complex, heterobimetallic complex, zero-gap cell electrolyzer

## Abstract

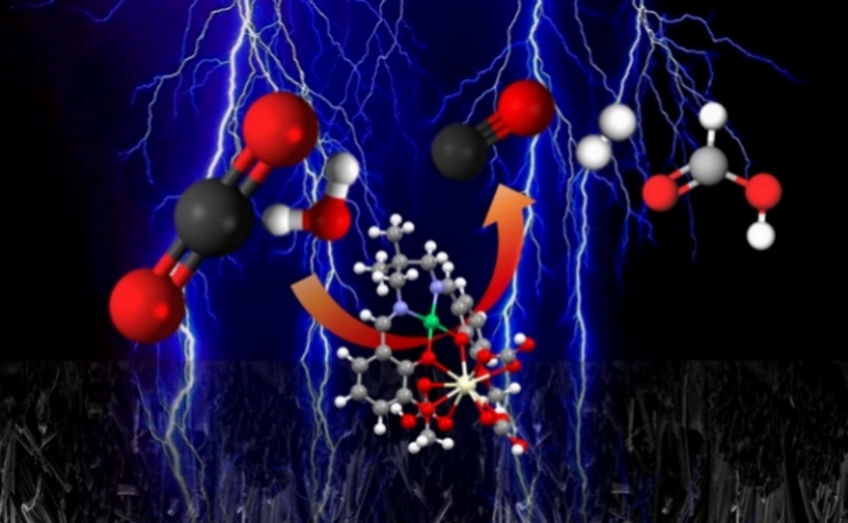

In this study, we propose a practical approach for producing a heterobimetallic Ni(II)–Ce(III) diimine complex from an extended salen-type ligand (H_2_L) to serve as an electrocatalyst for CO_2_ reduction and demonstrate an outstanding overall efficiency of 99.6% of the cerium–nickel complex and integrate it into applicable cell assemblies. We optimize not only the catalyst, but the operational conditions enabling successful CO_2_ electrolysis over extended periods at different current densities. A comparison of electrochemical behavior in H-cell and zero-gap cell electrolyzers suggests potential applications for industrial scale-up. In the H-cell electrolyzer configuration, the most elevated efficiency in CO production was achieved with a selectivity of 56.96% at −1.01 V vs RHE, while HCOO^–^ formation exhibited a selectivity of 32.24% at −1.11 V vs RHE. The highest TON was determined to be 14657.0 for CO formation, followed by HCOO^–^ with a TON of 927.8 at −1.11 V vs RHE. In the zero-gap electrolyzer configuration, the most efficient setup toward CO production was identified at a current density (CD) of 75 mA cm^–2^, a flow rate of 10 mL min^–1^, operating at 60 °C and utilizing a low KOH concentration of 0.1 M to yield a maximum faradaic efficiency (FE_CO_) of 82.1% during 24 h of stable electrocatalysis.

## 1 Introduction

The reduction of CO_2_ is pivotal in attaining climate neutrality and mitigating energy challenges.^1−7^ The development of efficient catalysts emerges as a crucial determinant, facilitating pathways to expedite the conversion of CO_2_ into valuable, environmentally friendly resources through electroreduction.^8^ The transformation of CO_2_ via electroreduction into industrial chemicals and usable fuels stands as a promising solution to these challenges. Despite the comprehensive study of traditional metallic catalysts, their inadequate durability, scarcity, high cost and substantial overpotentials hinder their practical application in real-world scenarios.^9,10^ Hence, one of the key challenges is the development of catalysts that can efficiently convert CO_2_ into high-value products such as syngas, methane, or ethylene. This requires catalysts with high selectivity and activity, as well as stability under the harsh conditions of electrosynthesis.^11^ Among many catalysts for the CO_2_ reduction reaction (CO_2_RR), Fe-based and Cu-based assemblies are one of the primary catalysts capable of converting CO_2_ into multicarbon products. However, the long-term stability of these catalysts at high current densities is often unsatisfactory, which hampers commercially relevant CO_2_ electrolysis.^12−16^ The OER (Oxygen Evolution Reaction) is a critical process often occurring at the anode during CO_2_ electroreduction, which plays a key role in balancing charge and maintaining efficient overall cell operation.^17^

In recent years, there has been growing interest in heterobimetallic catalysts due to their unique properties and potential for enhancing the selectivity and activity of CO_2_ electroreduction.^18−21^ It has been proven that synergistic effects between two different metal centers in a heterobimetallic catalyst can lead to improved catalytic performance compared to single-metal catalysts.^18−21^ Here, we investigate the electrocatalytic performance of a heterobimetallic Ni–Ce complex for CO_2_ reduction. By leveraging the complementary properties of nickel and cerium, we developed a catalyst that exhibits high selectivity and activity for the conversion of CO_2_. In addition, the use of Schiff-base ligands with alkoxy groups in the cerium–nickel complex introduces bicompartmental ligands, facilitating coordination with various metal ions. Our heterobimetallic complex is applied for the first time as a catalyst for electrosynthesis from CO_2_ feedstock. The combination of Ni and Ce efficiently activates the O=C=O bonds, where cerium with its Lewis acidic property pivotally shuttles CO_2_/bicarbonate to the reactive Ni center, thus considerably enhancing the electroreduction process.^22−26^ Comparisons with bare ligand and nickel-ligand systems highlight the catalytic synergy in the nickel–cerium complex, emphasizing the indispensable role of cerium in optimal CO_2_ electrocatalysis. Through a combination of electrochemical and spectroscopic techniques, we gain insights into the CO_2_ reduction process on the Ce–Ni molecule and optimize its performance for practical CO_2_ electroreduction applications. Furthermore, we explore the stability and durability of the Ce–Ni complex under prolonged electrochemical conditions to assess its potential for industrial-scale CO_2_ conversion technology.^27−29^ Meticulous electrochemical characterizations in both H-cell and zero-gap cell configurations reveal the cerium–nickel compound’s ability to produce diverse carbon-based products, showcasing its versatility. These promising outcomes position the cerium–nickel complex as a valuable electrocatalyst with significant implications for industrial applications in CO_2_ electrocatalysis.

## 2 Experimental Section

### 2.1 Synthesis of the Heterobimetallic Cerium-Nickel Complex

**Scheme 1:**
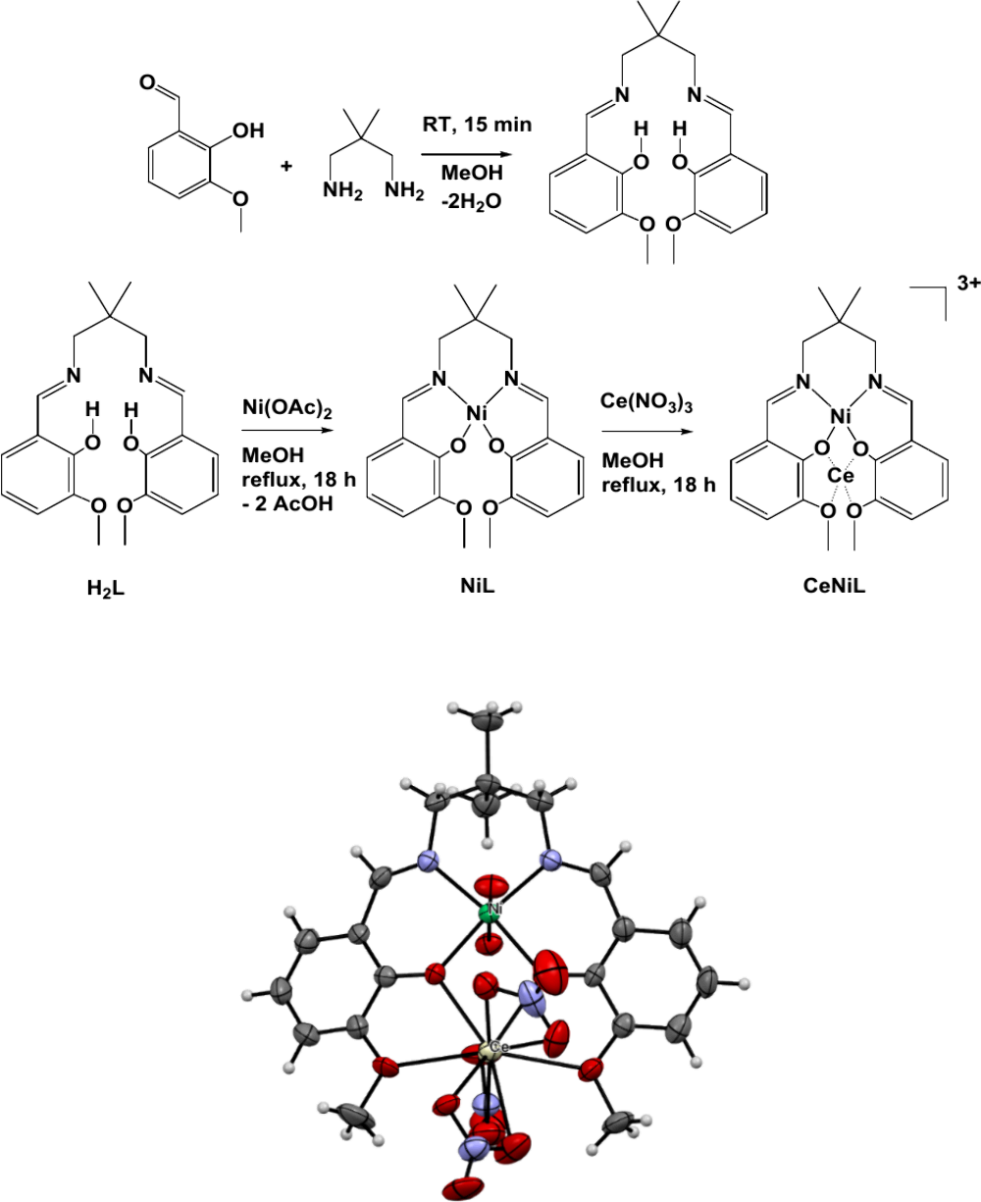
(Top) One-Step Synthesis of the Salen-Type Ligand**H**_**2**_**L**, Monometallic Complexes**NiL** and the Heterobimetallic Congener**CeNiL,** the NO_3_^–^ Anions of Compound**CeNiL** as well as the Crystal Water Molecules of the Applied Ni and Ce Salt Are Omitted in the Drawings for the Sake of Simplicity; (Bottom) Molecular Structure of Dinuclear**CeNiL** as Determined by Single Crystal X-ray Diffraction Analyses, the Axial Ligands That Are Linked to the Ni Center Stem from the Crystal Water in the Applied Nickel Precursor, and the Thermal Ellipsoids Were Drawn up to the 50% Probability Level and the H Atoms Are Omitted for Clarity

The synthesis of all three compounds, namely the H_2_L salen ligand (**L**), **NiL**, and **CeNiL**, involved the application of facile protocols sourced from the literature (Scheme 1 and Supporting Information).^30^

### 2.2 Materials and Reagents

Heterobimetallic cerium–nickel complex electrocatalyst inks were prepared by combining 2 mg of the electrocatalyst with 2 mg carbon black in 2 mL of methanol. To enhance the adhesion of the ink, 20 μL of a 5 wt % Nafion 117 solution (Sigma-Aldrich) was incorporated as a binder. The resulting catalytic inks underwent sonication for 30 min and were subsequently sprayed onto a 1 × 1 cm surface of carbon paper (TGP-H-60, Thermo scientific) for H-cell characterization, whereas for zero-gap cell measurements, 8 cm^2^ gas diffusion layers (GDE, CeTech) were applied (W1S1011-365 μm) and fully dried under vacuum overnight.

### 2.3 Characterization Methods

The catalytic loading was determined by weighing before and after spray-coating as 0.2 mg cm^–2^. Note that for XPS characterization the GDLs were prepared similarly only without the addition of Nafion in order to avoid suppression of other elements intensity by mainly carbon and oxygen. The electrochemical properties of the electrocatalysts were systematically examined through both homogeneous and heterogeneous approaches. Homogeneous electro-characterization was specifically employed to assess catalyst responsiveness to CO_2_ and involved cyclic voltammetry measurements. Glassy carbon served as the working electrode in 10 mL of acetonitrile with 0.1 M TBAPF_6_ as the supporting electrolyte, employing a scan rate of 30 mV s^–1^. Conversely, heterogeneous measurements were conducted to elucidate real electrochemical phenomena that could be encountered in scaled up applications. Here, linear sweep voltammetry (LSV) was performed in 0.1 M CsHCO_3_ aqueous electrolyte, as H_2_ formation was lowest in the case of CsHCO_3_. Initially, Ar gas (99.99%) was purged for 15 min through the CsHCO_3_ solution to remove air. The experiments were then carried out in 0.1 M CsHCO_3_ solution saturated with gaseous CO_2_ (99.99%) approximately for 1 h at a flow rate of 10 mL min^–1^ until the pH of the saturated solution reached 6.8. Cyclic voltammetry (CV) measurements under heterogeneous conditions were conducted to study the electrocatalytic effciency toward CO_2_ reduction reaction (e-CO_2_RR) in an aqueous electrolyte solution. The nickel–cerium–ligand assembly (**CeNiL**) was physisorbed on carbon paper as supportive electrode with an effective loading of 0.2 mg cm^–2^ and tested in a three-electrode configuration with CeNiL as working, Ag/AgCl/1 M KCl as reference and platinum wire as counter electrode. Cyclic voltammetry measurements were performed under Ar (red) and CO_2_ (blue) in 0.1 M CsHCO_3_ at pH 6.8 electrolyte solution. All heterogeneous electrochemical measurements were carried out in an H-type cell, where compartments were separated by a Nafion membrane, unless otherwise noted. The reference electrode was Ag/AgCl (with saturated KCl as the filling solution), and a Pt wire served as the counter electrode. Before measurements, the electrolyte solution (0.1 M CsHCO_3_) was preferentially purged with CO_2_ for 1 h at a flow rate of 50 mL min^–1^ and then bubbled continuously with CO_2_ at 10 mL min^–1^ during the test. Potentiostatic chronoamperometry (CA) in an H-type cell was conducted to measure the consumed electrons during electrosynthesis in coulomb by integration of the current over time. Throughout the electrolysis, CO_2_ gas was introduced into the cathodic compartment at a flow rate of 10 mL min^–1^ to maintain a CO_2_-saturated environment. The voltage on the working electrode was incrementally adjusted, ranging from −0.61 to −1.31 V vs RHE, and held steady for 1 h with stirring at each potential to record the corresponding chronoamperometric curve. The electrochemical active surface area (ECSA, cm^2^) was calculated by double-layer capacitance *C*_DL_, which was measured by conducting CV within a 100 mV window centered at 0.78 V vs RHE. All potentials were eventually transformed to the reversible hydrogen electrode reference through the following relationship:
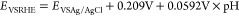
1

The different current densities (*i*_c_, mA cm^–2^) were plotted as a function of scan rate (*v*, mV s^–1^) with a slope equal to the *C*_DL_ (μF cm^–2^). The ECSA can be obtained by comparing the correlation *C*_DL_ (μF) to a smooth planar surface (*C*_REF_, μF cm^–2^) which was often assumed to be 40 μF cm^–2^ following these equations:


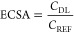
2


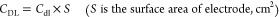
3

A comprehensive structural characterization was conducted using NMR, FTIR, UV–vis, and XPS techniques, verifying the proposed catalytic structure (Figures S1–S12). **NiL** and **CeNiL** were deposited on carbon paper through drop casting using a methanol mixture and underwent X-ray photoelectron spectroscopy (XPS) analysis both before (see Figures S3 and S4) and after the electroreduction (refer to Figures S5–S7). The XPS survey scans encompassed the corresponding Ni 2p, Ce 3d, N 1s, O 1s binding energy regions (see Figures S3–S7). In the Ni 2p3/2 region, the main peak at 856.3 eV is situated at a typical nickel(II) position, while the primary peaks for N 1s and O 1s are at 399.9 and 532.55 eV, respectively.^11,18^ The XPS measurements further revealed the presence of cerium mainly as Ce^3+^ and to a minor extent as Ce^4+^.^19−21^ The Ni 2p region partially overlaps with the one from Ce 3d, adding complexity to the analysis. However, the XPS scans demonstrate that the catalyst remains stable throughout the course of electrocatalysis (Figures S6 and S7). All zero-gap cell experiments related to CO_2_ electroreduction were conducted using an electrochemical configuration as illustrated in Figure S39. The cathode gas diffusion electrode (GDE), prepared with a catalyst (geometric active area of 1 mg cm^–2^), was separated from the anode by an anion exchange membrane (PiperION A40-HCO_3_). The membrane was conditioned overnight in 1 M KOH before being washed with Milli-Q water before electrolysis. The anode employed a Ti fleece with a loading of 1 mg cm^–2^ IrO_2_. A liquid electrolyte (0.1 M KOH) was introduced into the anolyte chamber on each side of the anion exchange membrane. Gaseous CO_2_ was fed into the cell behind the cathode GDE and diffused into the catalyst layer. Utilizing a temperature-controlled humidifier, the relative humidification of the CO_2_ gas was adjusted based on the applied current density. For each CO_2_ reduction experiment, fresh electrolyte was prepared, and it was circulated through the electrochemical cell using peristaltic pumps at a rate of 10 mL min^–1^. An automatic mass flow controller maintained the flow of the input CO_2_ (99.99%) at 100 sccm throughout each experiment.

## 3 Results and Discussion

### 3.1 Homogeneous and Heterogenous Electrocatalysis Experiments of CeNiL Complex

**Figure 1:**
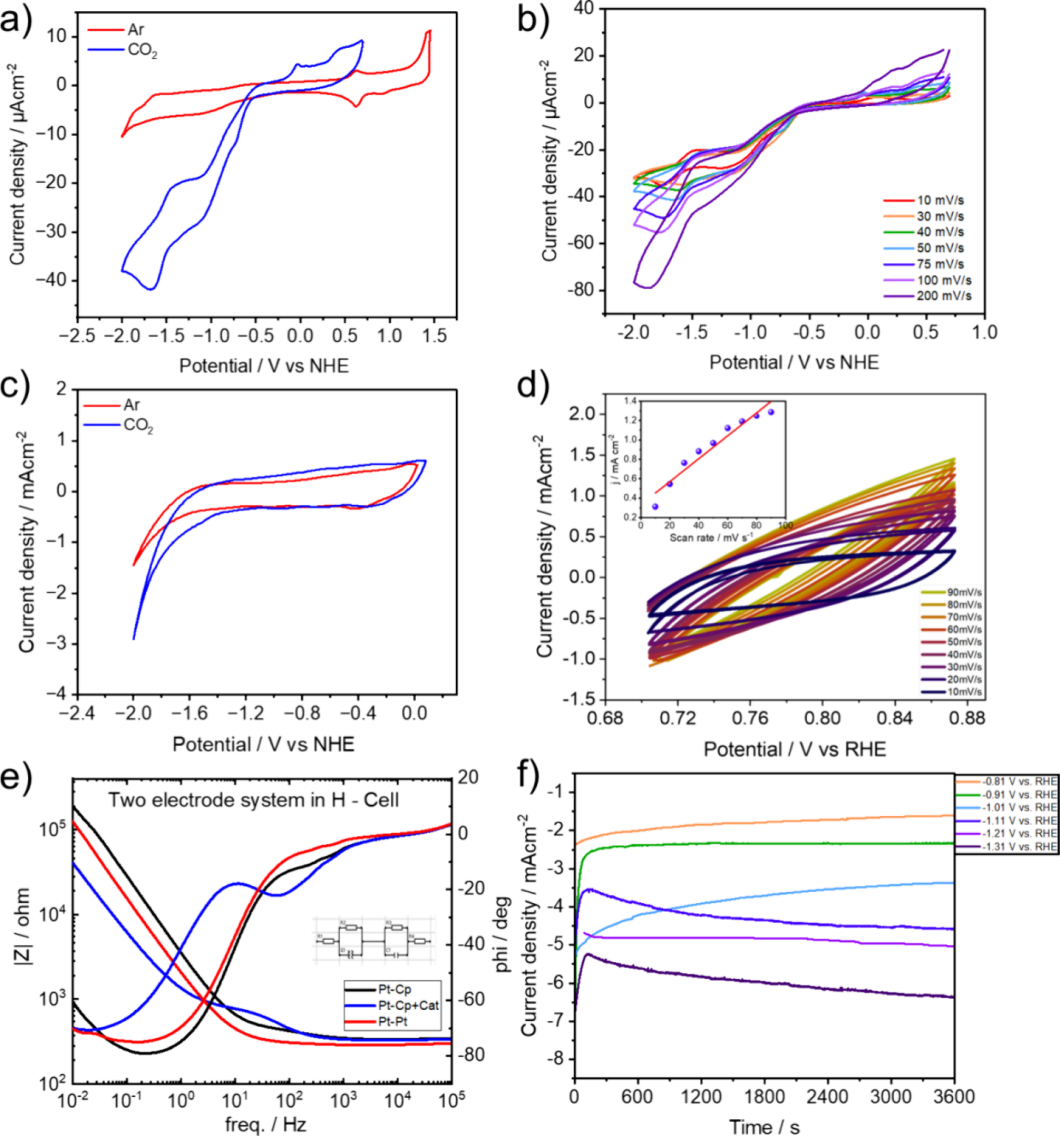
(a) Comparison of cyclic voltammograms of **CeNiL** dissolved in acetonitrile under argon and CO_2_ containing 0.1 M TBAPF_6_ as supporting electrolyte with glassy carbon as working, platinum wire as counter and nonaqueous pseudo-Ag/AgCl as reference electrode with a scan rate of 30 mV s^–1^. (b) CV curves of **CeNiL** dissolved in acetonitrile under CO_2_ containing 0.1 M TBAPF_6_ as supporting electrolyte. (c) CV curves at a scan rate of 30 mV s^–1^**CeNiL**/CB WEs with Ar and CO_2_ saturated 0.1 M CsHCO_3_ obtained in a H-cell. (d) Cyclic voltammograms of **CeNiL**/CB catalyst at different sweep rates of 10–90 mV s^–1^ from 0.70 to 0.87 V vs RHE in 0.1 M CsHCO_3_. Insert: a linear plot of capacitive current versus scan rate. (e) Bode plot recorded via electrochemical impedance spectroscopy in the frequency range of 1 × 10^–1^ Hz to 1 × 10^5^ Hz with a perturbation amplitude of 10 mV. (f) Cell current vs time plot at different half-cell potentials (V vs RHE).

The electrochemical characteristics of 1 mM **CeNiL** in acetonitrile were explored through CV measurements, in which glassy carbon served as the working electrode with 0.1 M TBAPF_6_ as the supporting electrolyte, and a scan rate of 30 mV s^–1^. As depicted in Figure 1a (red curve) and b, distinct one-electron redox peaks appeared at −1.0 and −1.62 V vs NHE, representing ligand and metal-centered electroreduction creating a ligand radical anion and Ni^1+^, as determined by in situ spectroelectrochemistry measurements (vide infra). The CV of the **CeNiL** on carbon paper was further investigated under Ar (Figure 1c, red curve) and CO_2_ (Figure 1c, blue curve) in aqueous conditions at pH = 6.8. In both cases, a marked increase in catalytic current was observed at a potential ranging from −1.62 to −1.75 V vs RHE, also corresponding to the reduction reaction of residual Ce^4+^ to Ce^3+^ (Figure 1c).^31^ Here the Lewis acid character of cerium facilitates the coordination of CO_2_/bicarbonate at these potentials and enhances the electroreduction of CO_2_ through the interplay with Ni, providing proton coupled electron transfers (PCET).

The first metal-centered oxidation at *E*_1/2_ = 0.625 V and *E*_p_ = 0.63 V vs NHE involves ring-centered oxidation where Ce^3+^ is oxidized to a Ce^4+^. In the cyclic voltammogram (Figure S21), “curve crossing” is observed, where the current on the return scan surpasses that on the forward scan. This phenomenon results from the increasing concentration of the active catalyst due to in situ generation, leading to higher catalytic current as the cyclic voltammetry progresses.^22,23,27,32−34^ Linear sweep voltammetry (LSV, Figure S16) was conducted in a 0.1 M CsHCO_3_ aqueous electrolyte, with CsHCO_3_ chosen for its amending effect on the electroreduction of CO_2_ (e-CO_2_R).^41^ In the CO_2_ saturated aqueous 0.1 M CsHCO_3_ medium, the cell current observed can stem not only from the electroreduction of CO_2_ but also from H_2_ gas generation, making it challenging to distinguish solely from LSV whether the observed peaks can be attributed to e-CO_2_RR or the hydrogen evolution reaction (HER). In our study, a pair of sharp and broad peaks were observed at *E*_cat_ = −1.02 V and *E*_cat_ = −1.62 V vs NHE for complex **CeNiL** in CO_2_-saturated media (Figure S16), indicating e-CO_2_RR and HER, respectively. The higher current density observed in the CO_2_-saturated environment compared to the Ar environment yet affirmed the assignment to the CO_2_ reduction reaction under those conditions.

The variation in total current density and scan rates was also recorded as CVs in CO_2_-saturated media (Figure 1b,d), showing an increase in current density with higher scan rates. Due to the slow nature of e-CO_2_RR, the peak was not observed at −1.02 V vs RHE beyond a certain scan rate (>60 mV s^–1^). A substantial increase in current density was observed upon CO_2_ saturation, accompanied by a broad wave in the linear sweep voltammetry beginning at −1.62 V vs NHE. This observation once again suggests that the immobilized **CeNiL** on carbon paper catalyzes the reduction of CO_2._^29,30,35−40^

As the reduction processes involve electron and proton transfers, electrochemical impedance spectroscopy (EIS) was employed to evaluate the charge-transfer resistance of the Ni(II)–Ce(III) diimine complex for carbon dioxide reduction electrolysis. This method was employed capturing the impedance spectrum within a frequency range of 10^5^–0.01 Hz, with a perturbation amplitude of 10 mV (Figure 1e). Initially, two platinum electrodes were employed in a single-cell configuration with the 0.1 M CsHCO_3_ aqueous electrolyte, serving as a control experiment to ascertain the electrolyte resistance. Subsequently, the setup was transitioned to an H-cell configuration with a Nafion membrane, enabling the determination and subtraction of the membrane resistance from the electrolyte resistance. Further experiments involved replacing one platinum electrode with a carbon paper electrode as the working electrode. Lastly, the carbon paper, coated with **CeNiL**, served as the working electrode for the complete electrochemical cell evaluation through EIS (Figure 1e). The resulting fitted and calculated impedance data, as well as resistance values for each cell component (electrolyte solution, membrane, carrier electrode) in the carbon dioxide reduction cell system are summarized in Table 1. The detailed characterization based on EIS revealed negligible losses in the applied electrochemical cells. Evidently, **CeNiL** demonstrated the lowest charge transfer resistance, indicating enhanced electrocatalytic kinetics (Figure 1e). Controlled potential electrolysis (CPE) was then performed at various potentials, ranging from −0.8 to −1.3 V vs RHE, over 1 h, resulting in current densities ranging from 6.5 to 21.45 mA cm^–2^ (see Figure 1f).

**Table 1:** Cell Parameters Extracted via Electrochemical Impedance Measurements

WE	CE	*R*_sol_/Ω	*R*_carrier_/Ω	*R*/_CeNi_Ω	*R*_Me_/Ω	*C*_CeNi_/F	CPE-T	CPE-P
Pt	Pt	3.2 × 10^1^	6.5 × 10^5^		2.9 × 10^2^		9.5 × 10^–5^	8.8 × 10^–1^
GC	Pt	3.2 × 10^1^	5.7 × 10^5^		2.9 × 10^2^		5.4 × 10^–5^	8.9 × 10^–1^
CeNi	Pt	3.2 × 10^1^	1.5 × 10^6^	3.8 × 10^2^	2.9 × 10^2^	8.7 × 10^–6^	2.4 × 10^–4^	8.7 × 10^–1^

**Figure 2:**
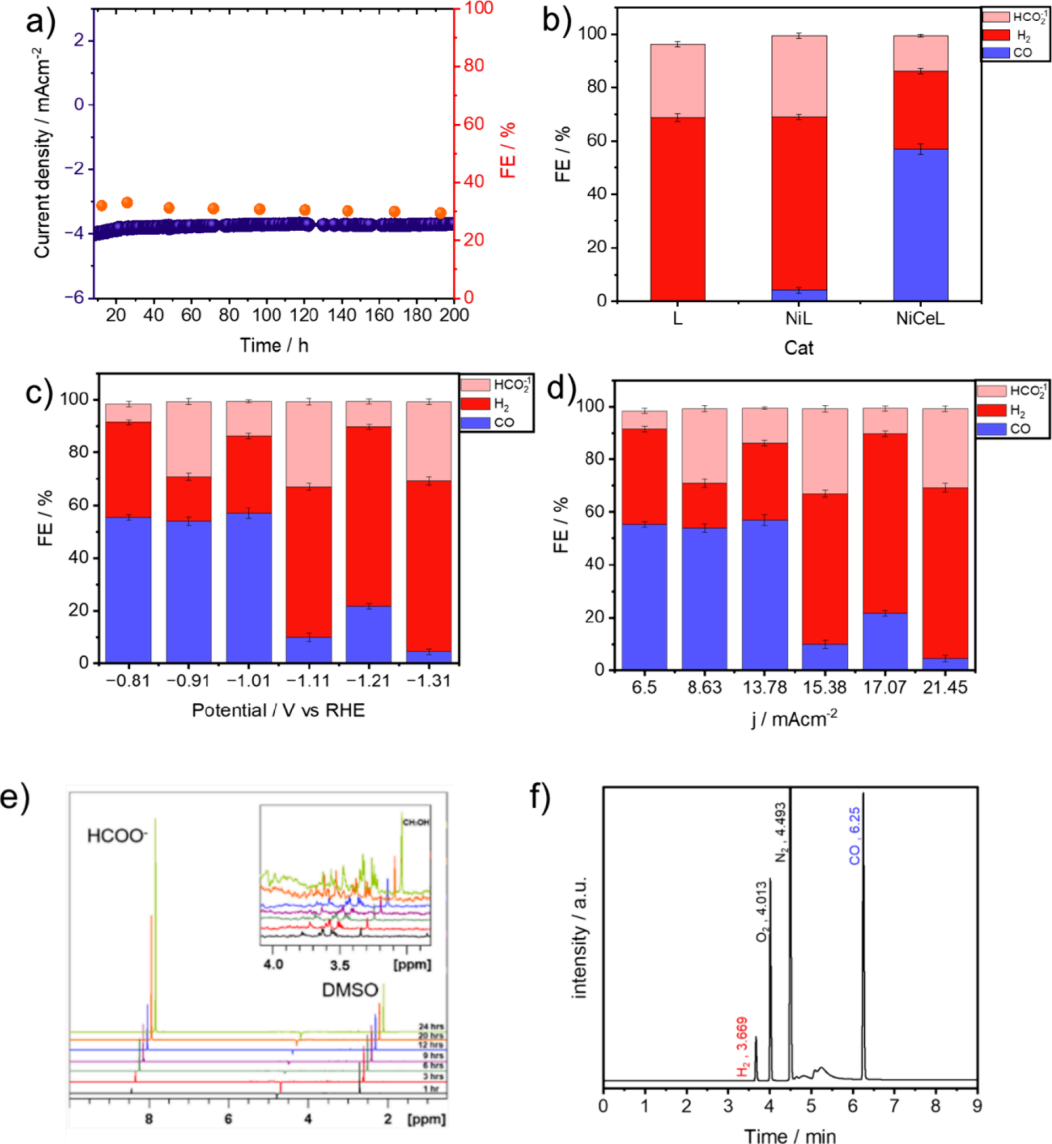
(a) Long-term stability test of **CeNiL**, cell current vs time at −1.11 V vs RHE and FE of formate. (b) Faradaic efficiencies of **CeNiL**/CB, **NiL**/CB, and **L**/CB catalyst for CO, H_2_, and HCOO^–^ obtained during 1 h electrolysis at −1.0 V vs RHE. (c) Faradaic efficiencies of **CeNiL**/CB for CO, H_2_, and HCOO^–^ obtained during 1 h electrolysis at each potential displayed. (d) Faradaic efficiencies of **CeNiL**/CB for CO, H_2_, and HCOO^–^ obtained during 1 h electrolysis at each current density displayed. (e) ^1^H NMR spectra of the liquid products formed after CO_2_ reduction at −1.21 V vs RHE by **CeNiL**/CB catalyst modified carbon paper electrode in 0.1 M CO_2_-saturated CsHCO_3_ solution. Insert: Production of traces of MeOH and propane-1,2-diol. (f) GC-BID chromatogram of gaseous products formed after CO_2_ reduction at −1.01 V vs RHE for 1 h by **CeNiL**/CB catalyst modified carbon paper electrode in 0.1 M CO_2_-saturated CsHCO_3_ solution.

Subsequently, electrocatalysis experiments were performed in a H-cell electrolyzer system (Figure S25). Product analysis, conducted via ^1^H NMR spectroscopy and gas-chromatography (Nexis GC-2030), revealed formate as the predominant liquid product, with CO and H_2_ identified as the gaseous compounds (see Figure 2b–f).

Over 24 h of CPE with **CeNiL**, formate was found to be the main liquid product with a faradaic efficiency (FE) of 32.24% at −1.1 V vs RHE (see Figure 2b–d). Furthermore, both the FE and current density remained relatively constant over 24 h of CPE (Figure 2e). CO exhibited faradaic efficiencies ranging from 4.31 to 56.96%, with product formation decreasing independently toward more negative potentials (see Figure 2c). At −1.1 V vs RHE, formate production was favored with a substantial increase in its FE (32.24%, Figure 2c). CO production was already observed at less negative potentials starting from −0.8 V (Figure 2c). Control measurements employing an unmodified salen ligand and nickel salen complex exhibited diminished product formation establishing the enhanced CO_2_ reduction capabilities of **CeNiL** (see Figure 2b–d). X-ray photoelectron spectroscopy (XPS) analysis of the coated carbon paper before and after the electrocatalysis reaction confirmed the stability of **CeNiL** throughout the CO_2_ reduction process (see Figures S3–S7). Methods for quantification of the respective gaseous and liquid compounds are described in Figures S28–S30. The faradaic efficiency of H_2_ production was 16.92% at *E*_cat_ = −0.91 V vs RHE, which increased to 85.93% at *E*_cat_ = −1.21 V vs RHE (see Figure 2c). The most elevated efficiency in CO production was achieved with a selectivity of 54.62% at −1.01 V vs RHE, while HCOO^–^ formation exhibited a selectivity of 21.8% at −1.11 V vs RHE. The highest TON was determined to be 14656.98 for CO formation, followed by HCOO^–^ with a TON of 927.75 at −1.11 V vs RHE. Notably, HCOO^–^ and CO displayed distinct TON and TOF values (see SI for calculation), indicating that **CeNiL** exhibits greater activity in the generation of CO. The stability assessment of **CeNiL** was conducted through an extended chronoamperometry at −1.11 V. As depicted in Figure 2a, neither the current density nor the faradaic efficiency (FE) of formate exhibited a noticeable decline over a duration of 6 days of electrolysis, showcasing the stability of **CeNiL**. In a final observation, it is however suggested that the electrocatalyst-electrolyzer architecture could be even further optimized regarding its stability as the system’s FE experienced a minor drop from 32% (32.24% HCOO^–^ at −1.11 vs RHE) to 29.2% after 200 h of reaction time. Furthermore, consistent results in both linear sweep voltammetry (Figures 2b and S14) and X-ray photoelectron spectroscopy (Figures S4–S7) before and after the prolonged reaction underscore excellent stability in activity. Poststability tests show that there were no significant changes in the valences of Ni and Ce, with Ni maintaining its Ni^2+^ state and Ce existing in the mixed states of Ce^3+^ and Ce^4+^ (vide supra, Figures S6 and S7). The ratio of Ce^3+^:Ce^4+^ yet showed a slight increase after the stability tests, attributable to the reduction process.

**Figure 3:**
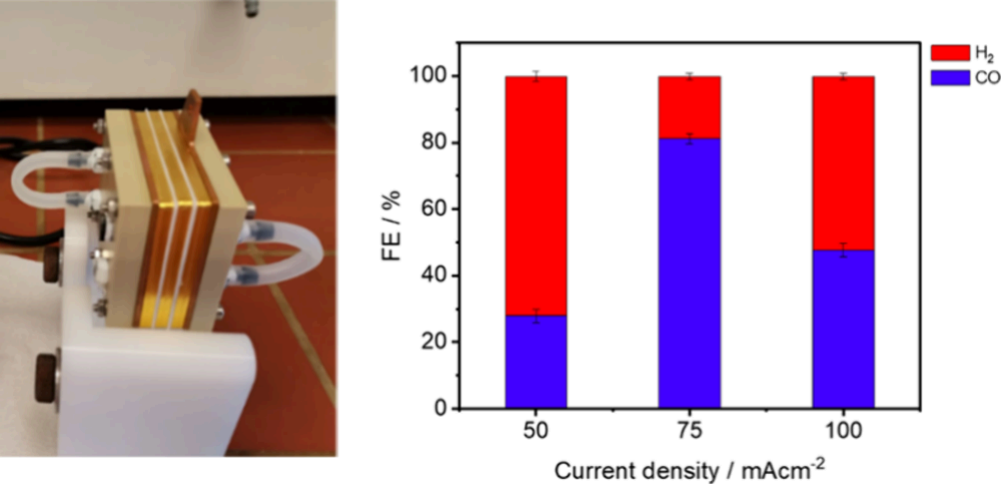
Performance of **CeNiL**/CB catalyst catalyst for the CO_2_ reduction to syngas in a home-built zero-gap electrolyzer cell stack at 50, 75, and 100 mA cm^–2^ at 60 °C after 24 h of electrolysis. All investigated GDEs possessed a catalytic loading of 0.5 mg cm^–2^ of active material.

In order to further probe the practical applicability of the investigated catalytic system, final measurements were conducted in a zero-gap cell electrolyzer. These assemblies are widely known to improve reaction efficiency by reducing the distance between the electrode and membrane, leading to lower resistance and better mass transfer, thus significantly enhancing achievable current densities.^44^ The here employed home-built zero-gap cell electrolyzer cell stack consisted of flow plates, sample and IrO_2_ electrodes, Teflon spacers, and a PiperION anion exchange membrane (40 μm) (Figure 3). In the pursuit of cost reduction for overall CO_2_ capture and conversion systems, attention is directed not only toward optimizing CO_2_ electrochemical reactors but also toward the capture and release of CO_2_ to the electrochemical cell. The previously considered inefficient KOH reduction, whose applicability was doubt, is now gaining attention as one of the most promising routes for developing an efficient integrated CO_2_ capture and conversion system involving the electrochemical reduction of CO_2_.^45^

Despite the promising findings in terms of cathodic and full cell electrical efficiency, the stability of the catalyst is crucial for commercial implementation. Commercial gas diffusion electrodes (GDEs) are known to suffer from stability issues, losing hydrophobicity and experiencing flooding over time. This behavior is exacerbated under pressure.^42^ For the investigated cerium nickel system the optimized operating parameters were determined to be a current density (CD) of 75 mA cm^–2^, a flow rate of 10 mL min^–1^, operating at 60 °C, and utilizing a low KOH concentration of 0.1M, yielding a maximum faradaic efficiency (FE_CO_) of 82.1% (Figure 3). These findings offer promising insights toward the implementation of finely designed KOH electrolyzers for the integrated capture and conversion of CO_2_.

### 3.2 Mechanistic Considerations of the CO_2_ Electroreduction Reaction

**Figure 4:**
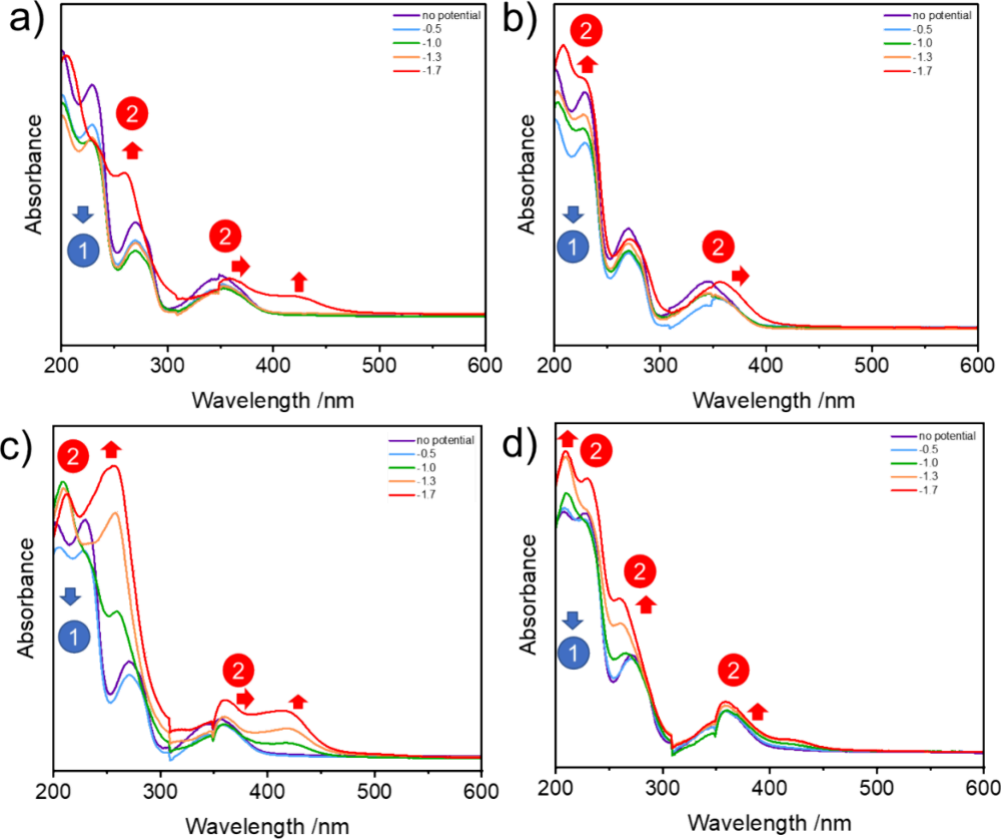
Spectroelectrochemistry of **CeNiL** with 0.2 M TBAPF_6_ in (a) ACN under Ar, (b) ACN under CO_2_, (c) ACN + 2% H_2_O under Ar, and (d) ACN + 2% H_2_O + CO_2_, at potentials from −0.5 to −1.7 V vs Ag/AgCl. **Process 1**: Ligand centered reduction; **Process 2**: Reduction of Ni^2+^ to Ni^1+^ and Ni-hydride formation. The kink at 350 nm stems from the lamp change of the employed instrument.

During operando electrochemistry-UV–visible spectroscopy (EC-UV–vis) experiments conducted in both aqueous and dry acetonitrile, within the potential range of −0.5 to −1.7 V vs Ag/AgCl, significant spectral changes were observed. The absorption intensity below 250 and 280 nm (as shown in Figure 4a,b) decreased, indicating a ligand-centered reduction process (process 1). When the potential exceeded −0.8 V vs Ag/AgCl in aqueous acetonitrile or −1.3 V vs Ag/AgCl in dry acetonitrile, the UV–visible spectrum exhibited a notable alteration, with a bathochromic shift and a significant increase in absorption at 260 and 420 nm. This indicated the formation of a new spectroscopically active species, suggesting a nickel-centered reduction process (process 2) that leads to the generation of the corresponding Ni^1+^ complex. In aqueous acetonitrile under argon, the spectral changes were more pronounced compared to those under CO_2_, presumably due to the more favored formation of a nickel-hydride complex (Figure 4c). In order to substantiate these hypotheses, experiments were conducted utilizing KC_8_ as chemical reducing agent for **CeNiL**. The resulting UV–vis spectra (Figure S32) in dry and aqueous acetonitrile resembled the ones obtained by electrochemical reduction, thus convincing that identical catalyst species are generated. ^1^H NMR spectroscopy of the chemically reduced catalyst in nondried CD_3_CN revealed strong paramagnetism and an intense signal at approximately −6 ppm (Figure S33), which is in the reported range of hydride signals for Ni(I) complexes.^43^

The resulting proposed electrocatalytic pathway 1 involves a ligand-centered reduction to form radical anions, which are catalytically active in producing HCOO^–^ and particularly H_2_. In contrast, the proposed electrocatalytic pathway 2 involves a metal-hydride process, similarly leading to the production of H_2_, formic acid/formate, but additionally small amounts of CO, if the ligand is solely metalated with nickel (compare Figure 2b). These two pathways operate at different reduction potentials: pathway 1 occurs at lower reduction potentials (≤−1.0 V vs NHE), whereas pathway 2 favors H_2_/formate evolution through hydride formation at higher reduction potentials (≥−1.0 V vs NHE). The introduction of cerium into the catalytic framework, however, beneficially alternates its behavior (Figure 2b). The cerium ion as strong Lewis acid is capable of coordinating to HCO_3_^–^/CO_2_ and hence accelerates the HCO_3_^–^ flux to the catalyst’s reactive center. Bringing the molecule of interest in spatial proximity to the reactive nickel hydride as well as lowering pH values (approximately 6.8) at the cathode surface shifting the HCO_3_^–^/CO_2_ equilibrium toward a higher CO_2_ concentration, it facilitates the hydrides interception by reacting with carbon dioxide instead of recombining with protons to form H_2_ gas. Considering the obtained faradaic efficiencies as well as the spectroscopic results from electrochemical and chemical reduction it is proposed that the synergistic effect of the complexed cerium ion enhances the kinetics of CO_2_ to CO conversion at low reduction potentials compared to the formation of H_2_ and formate.

## 4 Conclusions

In summary, this investigation focuses on employing a molecular, heterobimetallic cerium–nickel catalyst to electrochemically reduce CO_2_ to CO in H-cell and zero-gap cell setups. This process achieved maximum faradaic efficiencies of 54.6% for CO (FE_CO_) and 21.8% for formate (FE_HCOO_^–^) in the H-cell setup with stable performance for 200 h. Electrocatalysis experiments in a zero-gap cell electrolyzer demonstrated a FE_CO_ of 82.1% at current densities of 75 mA cm^–2^.

Our study demonstrates that the explored **CeNiL** complex exhibits high selectivity for CO production from CO_2_ at low reduction potentials. This efficiency is enabled by the strongly Lewis acidic cerium ion in spatial proximity to the nickel center, substantially accelerating the CO_2_ to CO reduction process. Overall, this research presents a potent method for directly generating CO through electrocatalytic CO_2_ reduction using a readily available **CeNiL**-based molecular catalyst. Future investigations in our laboratory will explore the use of homobimetallic or heterobimetallic salen-type catalysts, which have the potential to exhibit superior activity toward electrocatalytic CO_2_ reduction, electrocatalytic N_2_ reduction and hydrogen evolution.

## Supporting Information Available

Synthetic procedures, structural (XPS, NMR, UV–vis, IR spectroscopy) and electrochemical characterization of the catalysts, product analysis, spectroelectrochemistry, chemical reduction experiments, and geometry optimization (PDF)
Crystallographic data (CIF)

## Supplementary Material

ae4c02132_si_001.pdf

ae4c02132_si_002.cif
