# Supplementary material for: Synergistic Interactions in a Heterobimetallic Ce(III)–Ni(II) Diimine Complex: Enhancing the Electrocatalytic Efficiency for CO2 Reduction
Source: ACS Appl Energy Mater. 2024 Oct 21;7(21):10052–60. doi: 10.1021/acsaem.4c02132 (PMC11558560; doi:10.1021/acsaem.4c02132)
Supplement: Supplementary file 1 — ae4c02132_si_001.pdf [file ae4c02132_si_001.pdf]

Supporting Information

Synergistic Interactions in a Heterobimetallic Ce(III)-Ni(II) Diimine Complex: Enhancing the Electrocatalytic Efficiency for CO_2 Reduction

Farzaneh Yari[a], Abdalaziz Aljabour[a], Houssein Awada[a], Jessica Michalke[b], Nidhi Kumari[c], Halime Coskun-Aljabour[a], Soumyajit Roy[c], Dominik Krisch[a,*] and Wolfgang Schöfberger[a,*]

[a] Institute of Organic Chemistry, Laboratory for Sustainable Chemistry and Catalysis (LSusCat), Johannes Kepler University (JKU), Altenberger Straße 69, 4040 Linz, Austria.

[b] Chair of Physical Chemistry, Montanuniversität Leoben, 8700 Leoben, Austria and Institute for Catalysis (INCA), Johannes Kepler University, 4040 Linz, Austria.

[c] Eco-Friendly Applied Materials Laboratory, Department of Chemical Sciences, Materials Science Centre, Indian Institute of Science Education and Research, Kolkata-741246, West Bengal, India.

*Corresponding authors: wolfgang.schoefberger@jku.at, dominik.krisch@jku.at

Keywords: CO_2RR, electrocatalysis, electrosynthesis, cerium-nickel complex, zero-gap-cell, electrolyzer

Content:

1. Synthesis of Ligand (H_2L) 2
2. Synthesis of heterobimetallic complex 3
3. Structural characterization of the catalysts (XPS, NMR, UV-vis, IR Spectroscopy) 6
4. Electrochemical characterization 14
5. Characterization by Electrochemical Impedance Spectroscopy (EIS)- Method 20
6. Product analysis by GC-BID 21
7. Product detection and quantification by ^{1}H-NMR 22
8. Spectroelectrochemistry and chemical reduction experiments 25
9. Geometry Optimization, Frequency Calculations (Infrared and Raman) 27
10. References 32

1. Synthesis of Ligand (H_2L)

The requisite binucleating ligand **H_2L** is readily assembled prior to use upon reaction of *ortho*-vanillin with 1,3-diamino-2,2′-dimethylpropane in methanol (Figure S1). Of note, this imine condensation is accomplished within less than one hour at room temperature (RT) and without the necessity of an exogeneous heating source. Furthermore, the experimental procedure is free from any laborious isolation and purification steps [1].

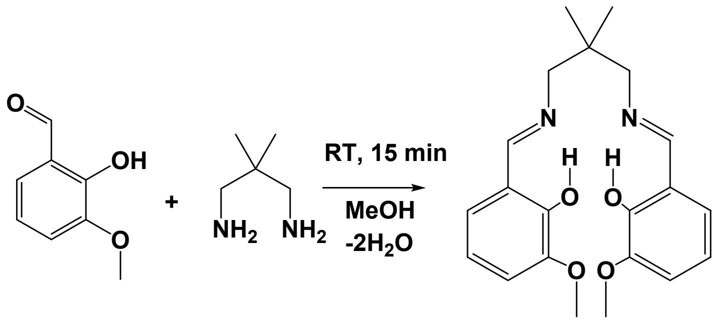

Figure S1. One-step synthesis of the salen-type ligand **H_2L** through reaction of MeO-functionalized salicylaldehyde A (o-vanillin) with a simple aliphatic diamine B. This straightforward imine condensation is carried out without any external heat sources. RT: room temperature.

2. Synthesis of heterobimetallic complex

The prepared multidentate ligand **H_2L** was used without further modifications to obtain the starting material for the solid composite catalysts (vide infra) in two steps. Metalation of the given chelating agent with $Ni(OAc)_2\cdot 4H_2O$ produced the neutral monometallic complex [NiL] while the consecutive reaction of the latter with $Ce(NO_3)_3\cdot 6\ H_2O$ neatly furnished the lanthanide-modied ionic compound **CeNiL** (Scheme 2). Crystals suitable for X-ray diffraction analysis were obtained upon slow diffusion of diethylether into a solution of [CeNiL] in acetone. Notably, it was found that the three NO_3^- counter anions were bound to the Ce^{3+} center[1].

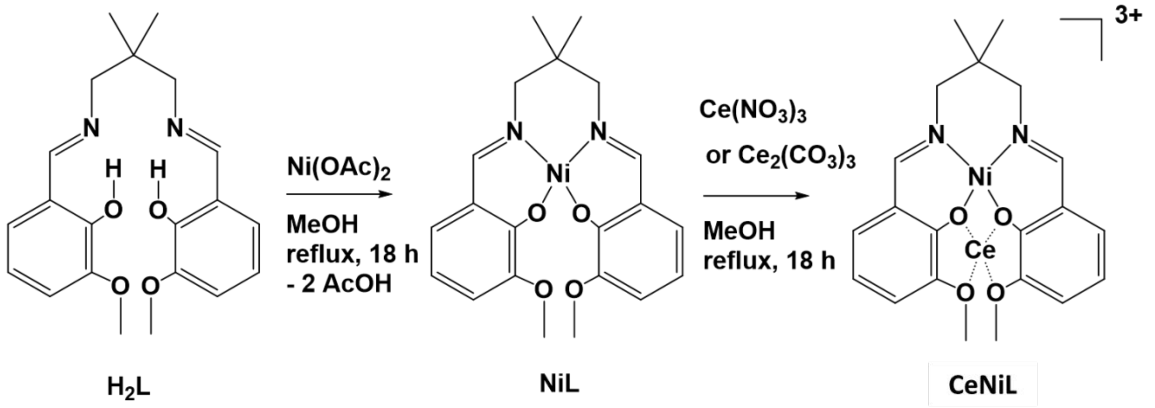

Figure S2. Syntheses of monometallic complexes **NiL** and the heterobimetallic congener **CeNiL** from the extended salen-type ligand **H_2L** using commercial and readily available $Ni(OAc)_2\cdot 4\ H_2O$ and $Ce(NO_3)_3\cdot 6\ H_2O$, respectively. The NO_3^- and CO_3^{2-} counter anion of compound **CeNiL** as well as the crystal water molecules of the applied Ni and Ce salt are omitted in the drawings for the sake of clarity.

Experimental Details

The catalytic loading was determined by weighing before and after spray-coating as 0.2 mg cm^{-2}. Note that for XPS characterization the GDLs were prepared similarly only without the addition of Nafion in order to avoid suppression of other elements intensity by mainly carbon and oxygen. The electrochemical properties of the electrocatalysts were systematically examined through both homogeneous and heterogeneous approaches. Homogeneous electro-characterization was specifically employed to assess catalyst responsiveness to CO_2 and involved cyclic voltammetry measurements. Glassy carbon served as the working electrode in 10 mL of acetonitrile with 0.1 M $TBAPF_6$ as the supporting electrolyte, employing a scan rate of 30 mV s^{-1}. Conversely, heterogeneous measurements were conducted to elucidate real electrochemical phenomena that could be encountered in scaled-up applications. Here, linear sweep voltammetry (LSV) was performed in 0.1 M $CsHCO_3$ aqueous electrolyte, as H_2 formation was lowest in the case of $CsHCO_3$. Initially, Ar gas (99.99%) was purged for 15 min through the $CsHCO_3$ solution to remove air. The experiments were then carried out in 0.1 M $CsHCO_3$ solution saturated with gaseous CO_2 (99.99%) approximately for 1h at a flow rate of 10 mL min^{-1} until the pH of the saturated solution reached 6.8 [2–4]. Cyclic voltammetry (CV) measurements under heterogeneous conditions were conducted, to study the electrocatalytic efficiency towards CO_2 reduction reaction (e-CO_2RR) in an aqueous electrolyte solution. The cerium-nickel complex (**CeNiL**) was physisorbed on carbon paper as a supportive electrode with an effective loading of 0.2 mg cm^{-2} and tested in a three-electrode configuration with **CeNiL** as working, Ag/AgCl/1M KCl as reference and platinum wire as counter electrode. Cyclic voltammetry measurements were performed under Ar (red) and CO_2 (blue) in 0.1 M $CsHCO_3$ at pH 6.8 electrolyte solution. All heterogeneous electrochemical measurements were carried out in an H-type cell, where compartments were separated by a Nafion membrane, unless noted otherwise. The reference electrode was Ag/AgCl (with saturated KCl as the filling solution), and a Pt wire served as the counter electrode. Before measurements, the electrolyte solution (0.1 M $CsHCO_3$) was purged with CO_2 for one hour at a flow rate of 50 mL min^{-1} and then bubbled continuously with CO_2 at 10 mL min^{-1} during the test. Potentiostatic chronoamperometry (CA) in an H-type cell was conducted to measure the consumed electrons during electrosynthesis in coulomb by integration of the current over time. Throughout the electrolysis, CO_2 gas was introduced into the cathodic compartment at a flow rate of 10 mL min^{-1} to maintain a CO_2-saturated environment. The voltage on the working electrode was incrementally adjusted, ranging from -0.61 to -1.31 V vs. RHE, and held steady for one hour with stirring at each potential to record the corresponding chronoamperometric curve. The electrochemical active surface area (ECSA, cm^2) was calculated by double-layer capacitance C_{DL}, which was measured by conducting CV within a 100mV window centered at

0.78 V vs. RHE. All potentials were eventually transformed to the reversible hydrogen electrode reference through the following relationship:

$$E_{vs\,\mathrm{RHE}} = E_{vs\,\mathrm{Ag/AgCl}} + 0.209\ \mathrm{V} + 0.0592\ \mathrm{V} \times \mathrm{pH}$$

The different current densities (i_c, mA cm^{-2}) were plotted as a function of scan rate (v, mV s^{-1}) with a slope equal to the C_{DL} (μF cm^{-2}). The ECSA can be obtained by comparing the correlation C_{DL} (μF) to a smooth planar surface (C_{REF}, μF cm^{-2}) which was often assumed to be 40 μF cm^{-2} following these equations: [2–4]

$$\mathrm{ECSA} = \frac{C_{DL}}{C_{REF}}$$

$C_{DL} = C_{dl} \times S$ (S is the surface area of electrode, cm^2)

A comprehensive structural characterization was conducted using NMR, FTIR, UV-Vis, and XPS techniques, verifying the catalytic structure (Figures S1-12). NiL and CeNiL were deposited on carbon paper through drop casting using a methanol mixture and underwent X-ray photoelectron spectroscopy (XPS) analysis both before (see Fig. S3 and S4) and after the electroreduction (refer to Fig. S5-7). The XPS survey scans encompassed the corresponding Ni2p, Ce3d, N1s, O1s binding energy regions (see Fig. S3-7). In the Ni 2p3/2 region, the main peak at 856.3 eV is situated at a typical nickel(II) position, while the primary peaks for N1s and O1s are at 399.9 eV and 532.55 eV, respectively [5,6]. The XPS measurements further revealed the presence of cerium mainly as Ce^{3+} and to a minor extent as Ce^{4+} [7–9]. The Ni2p region partially overlaps with the Ce3d, adding complexity to the analysis. However, the XPS scans demonstrate that the catalyst remains stable throughout the course of electrocatalysis (see Fig. S6,7).

All zero-gap cell experiments related to CO_2 electroreduction were conducted using an electrochemical configuration, as illustrated in Figure S36. The cathode gas diffusion electrode (GDE), prepared with a catalyst (geometric active area of 1 mg cm^2), was separated from the anode by an anion exchange membrane (PiperION A40-HCO3). The membrane was conditioned overnight in 1 M KOH before being washed with Milli-Q water before electrolysis. The anode employed a Ti fleece with a loading of 1 mg cm^{-2} IrO_2. A liquid electrolyte (0.1 M KOH) was introduced into the anolyte chamber on each side of the anion exchange membrane. Gaseous CO_2 was fed into the cell behind the cathode GDE and diffused into the catalyst. Utilizing a temperature-controlled humidifier, the relative humidification of the CO_2 gas was adjusted based on the applied current density [3,4]. For each CO_2 reduction experiment, fresh electrolyte was prepared, and it was circulated through the electrochemical flow cell using peristaltic pumps at a rate of 10 mL min^{-1}. An automatic mass flow controller maintained the flow of the input CO_2 (99.99%) at 100 sccm throughout each experiment.

3. Structural characterization of the catalysts (XPS, NMR, UV-vis, IR Spectroscopy)

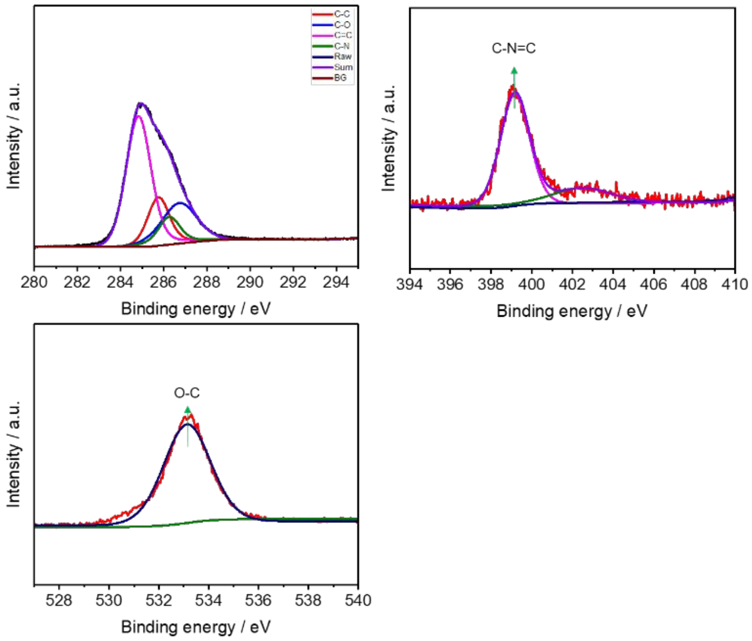

Figure S3. XPS Analysis of the ligand $\mathbf{H_2L}$ a) survey scan, b) N1s of salen with a binding energy of 399.9 eV and c) O1s with a binding energy of 532.55 eV.

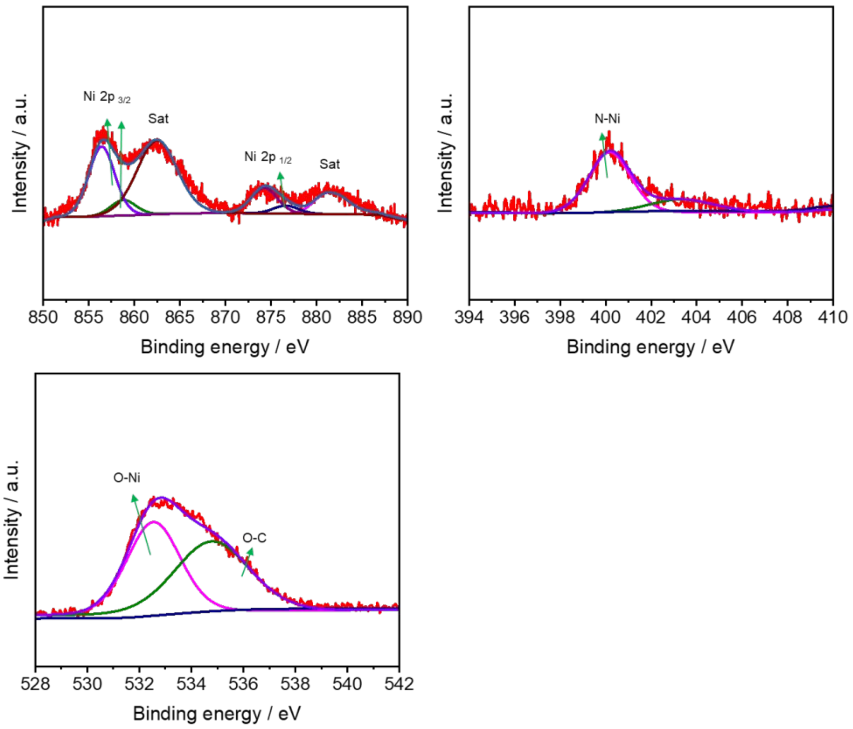

Figure S4. XPS Analysis of the **NiL**, Ni 2p3/2 spectra with BE of 856.3 eV and Ni 2p1/2 with BE of 873.5 eV**.**

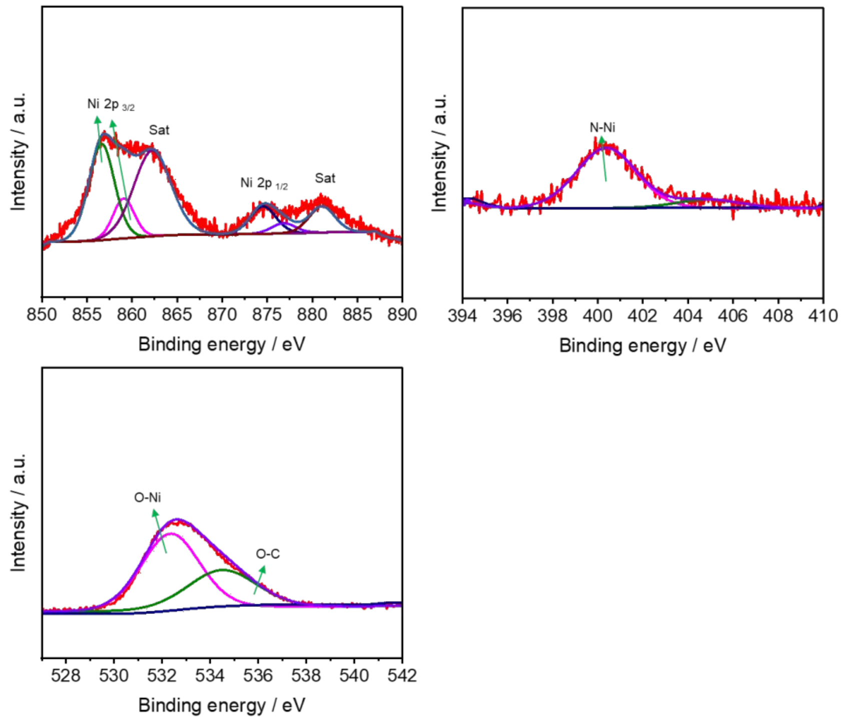

Figure S5. XPS Analysis of the **NiL**/CB after 24 h of electrolysis at -1.01 vs RHE.

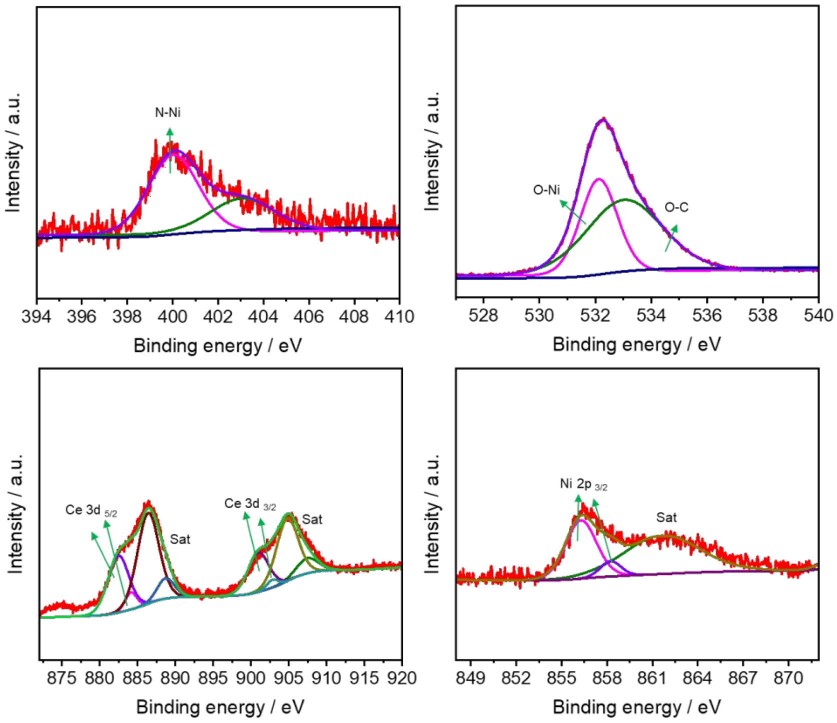

Figure S6. XPS Analysis of the **CeNiL** complex.

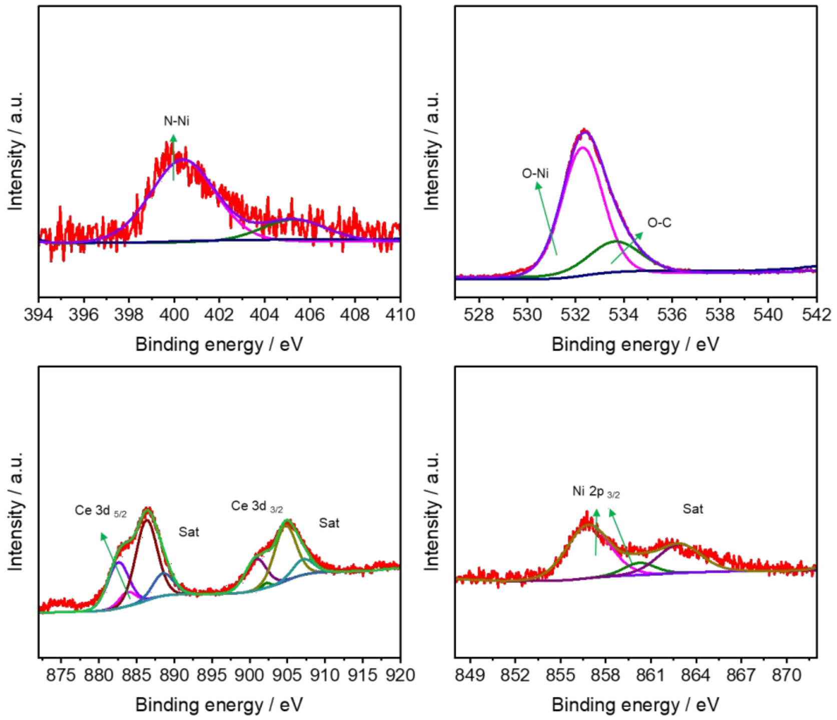

Figure S7. XPS Analysis of the **CeNiL**/CB after 24 hours of electrolysis at -1.21 vs RHE.

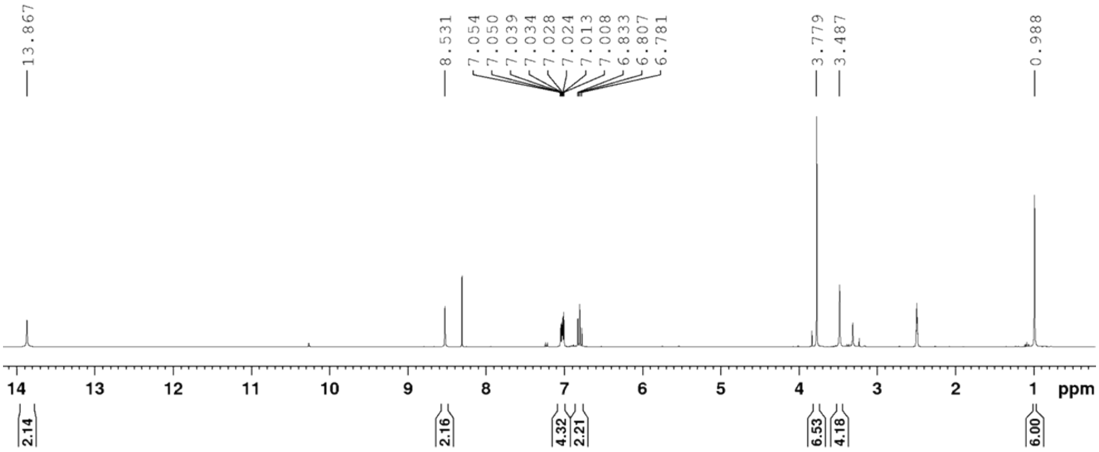

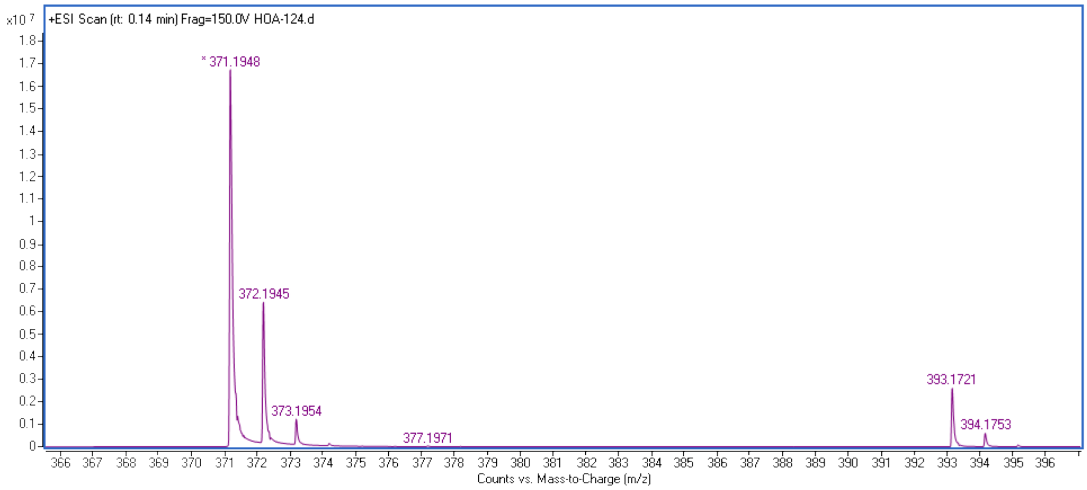

Figure S8, A) ^{1}H-NMR of the ligand **H_2L** (300 MHz, DMSO, 25 °C): δ= 13.87 (s, 2H, -OH), 8.53 (s, 2H, -N=CH-), 7.04 (m, 4H, aromatic), 6.83 (m, 2H, aromatic), 3.78 (s, 6H, -OMe), 3.49 (s, 4H, -CH_2-), 0.99 (s, 6H, -CH_3); B) HR-MS of [L]. m/z: calcd. for $C_{21}H_{27}N_2O_4^+$: 371.1971; found $[M+H]^+$: 371.1948

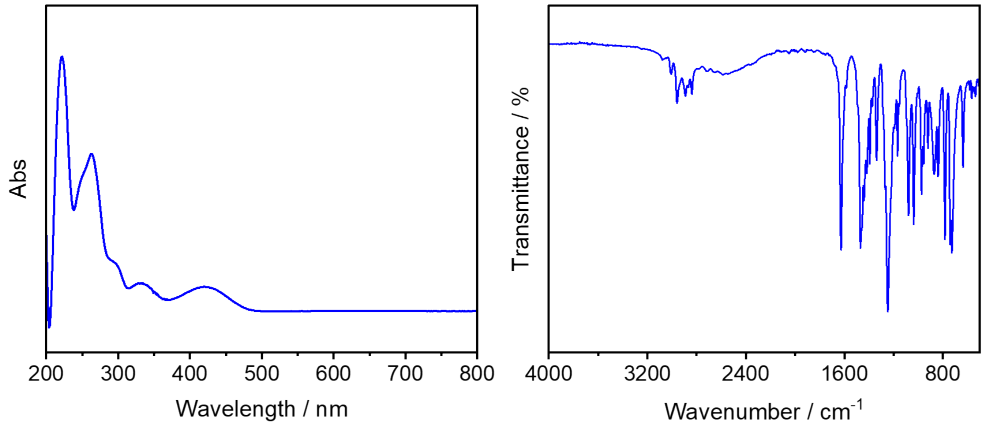

Figure S9. A) UV-vis of ligand $\mathbf{H_2L}$, λ_{max} nm: 222, 263, 334, 421. B) FT-IR of ligand

UV-vis Spectroscopy:

The spectrum of $\mathbf{H_2L}$ exhibits four bands at 421, 334, 263, and 222nm. The first two bands seem to be splitter band of n-π* transition, and other two bands are due to π- π * transitions, respectively.

FT-IR Spectroscopy:

Of particular significance is the peak at 1635 cm^{-1} attributed to C=N vibration in the $\mathbf{H_2L}$ ligands, which disappears upon hydrogenation, and a new peak at 3286 cm^{-1} attributed to N–H vibration is substitutionally observed in the spectrum of $\mathbf{H_2L}$. It is worthy to point out that the broad peaks in the 2700–2500 cm^{-1} region are due to the intra molecular hydrogen bonding between phenolic–OH and -NH resulting from the more flexible $\mathbf{H_2L}$ ligands. Comparison of the band positions of metal complexes with ligands reveals the following facts: (i) the broad peak ascribed to hydrogen bonding is absent,indicating the destruction of the hydrogen bond followed by the coordination of azomethine nitrogen and phenolic oxygen after deprotonation and (ii) the *v*(CN) stretch at 1635 cm^{-1} shifts to lower frequency in complexes, indicating the coordination of azomethine nitrogen. The coordination of –NH groups cannot be ascertained due to the appearance of broad band of water at about 3400 cm^{-1}.The appearance of two or four bands in the low frequency region (580–400 cm^{-1}) indicates the coordination of phenolic oxygen and azomethine nitrogen or secondary amine groups. However, upon anchorage of ligand or metal complex, these bands become vague due to the appearance of broad and strong bands at 1086, 807 and 402 cm^{-1} assigned to the framework as well as primary amine vibrations at 3200 cm^{-1}. Only some weak peaks at 1600–1300 cm^{-1} can be equivocally ascribed to the existence of organic ligands in the hybrid materials.

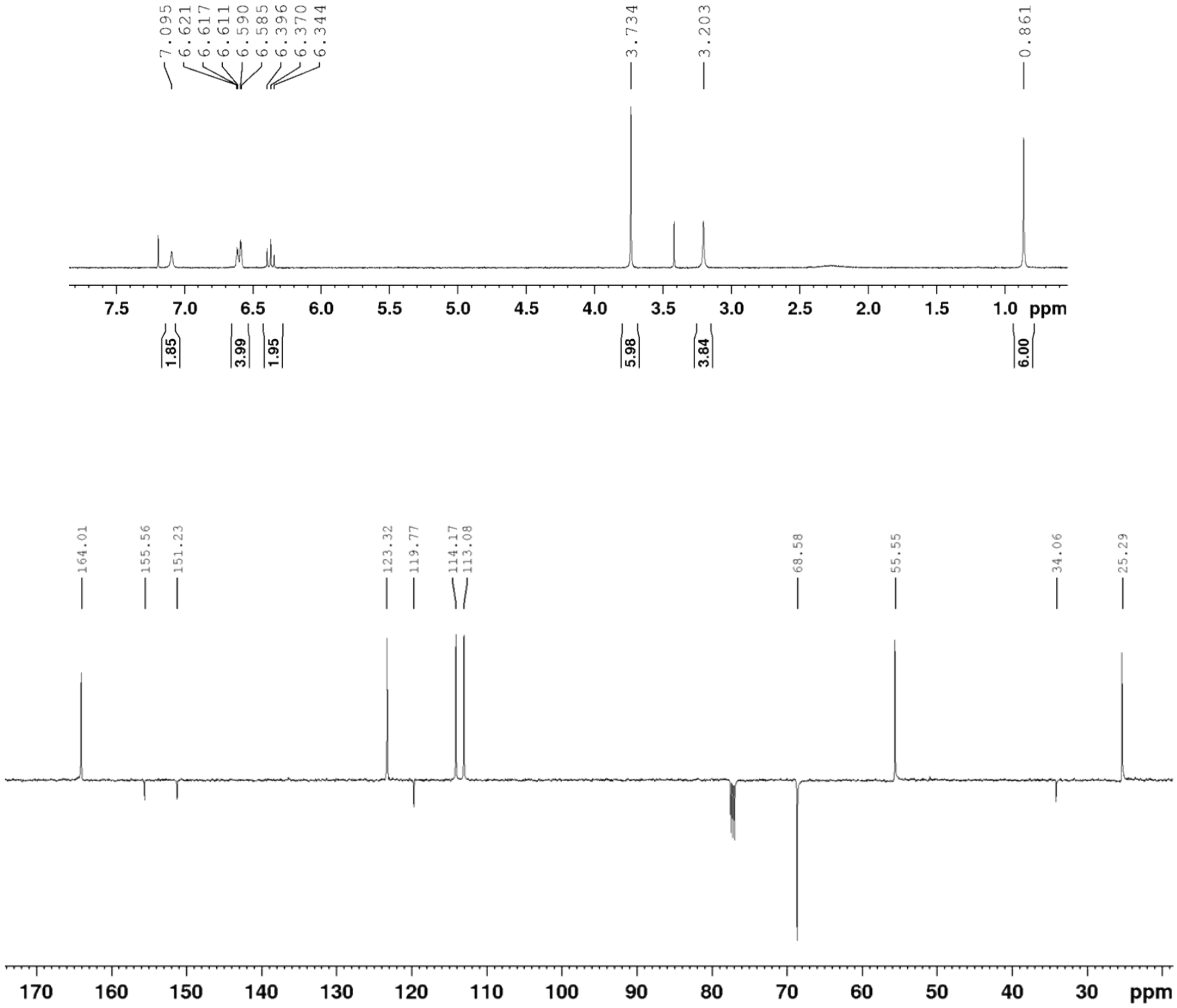

Figure S10. ^{1}H-NMR of **[NiL]** (300 MHz, $CDCl_3$, 25 °C): δ= 7.10 (s, 2H, N=CH), 6.59 – 6.62 (m, 4H, aromatic), 6.34 – 6.40 (m, 2H, aromatic), 3.73 (s, 6H, OMe), 3.20 (s, 4H, CH2), 0.86 (s, 6H, CH_3); B) ^{13}C-NMR of [NiL] (125 MHz, $CDCl_3$, 25°C): δ_C, ppm: 164.0 , 155.6 , 151.2, 123.3, 119.8, 114.2, 113.1, 68.6, 55.6, 34.1, 25.3.

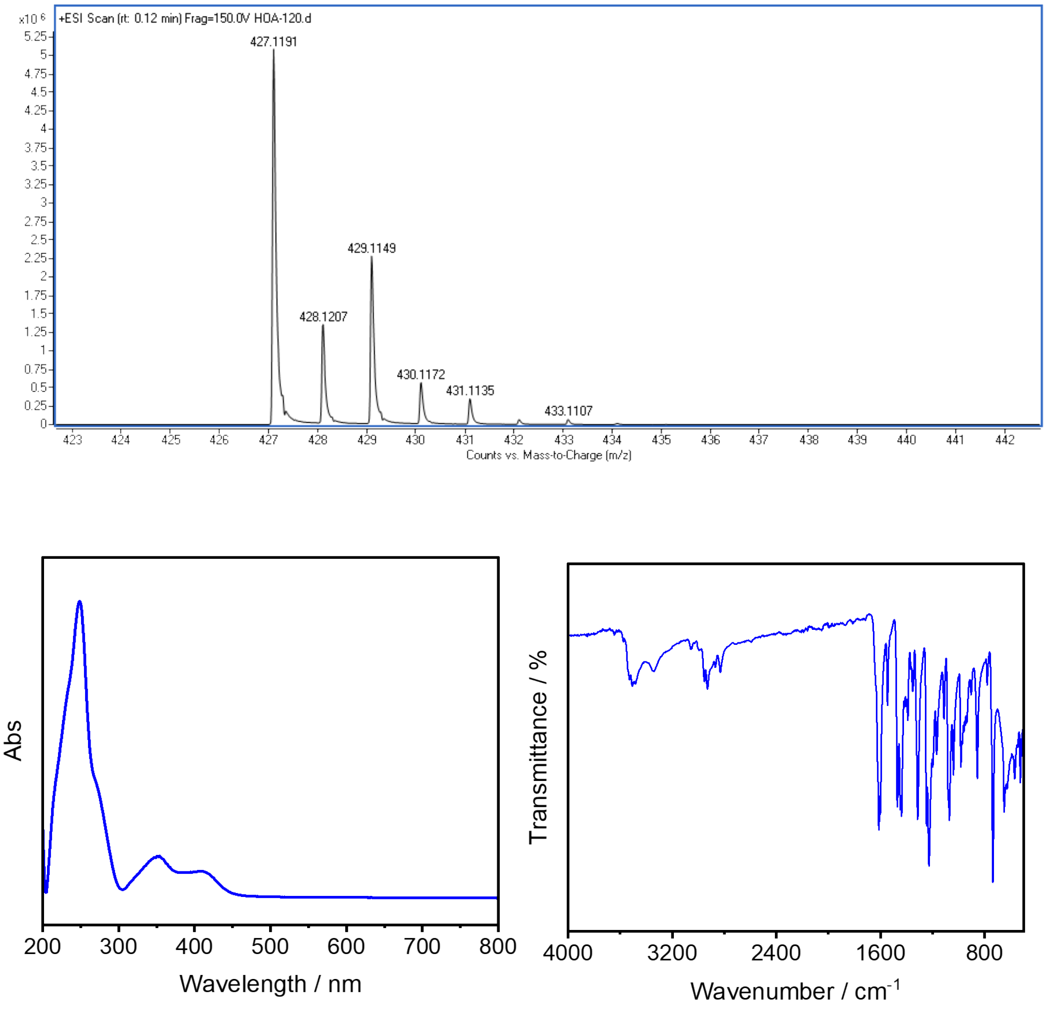

Figure S11. Nickel complex **NiL**. HR-MS of **NiL**. m/z: calcd. for $C_{21}H_{25}N_2NiO_4^+$:427.1168; found $[M+H]^+$: 427.1191. UV-Vis spectrum of **NiL** λ_{max} nm: 248, 354, 412 and FT-IR spectrum of **[NiL]**.

The UV−vis absorption spectrum show peaks at 248, 354, and 412 nm which are characteristic of the [Ni(salen)] complex **NiL**; (ii) the FTIR spectra display bands in the region of 2800− 3100 cm^{-1} (which corresponds to aliphatic and aromatic stretching of -C−H and =C−H, respectively), bands between 1450 and 1600 cm−1 (typical of C−C stretching in aromatic ring), and bands at 1624 and 1127 cm−1 (related to stretching vibrations of the azomethine and phenolics groups, both coordinated with nickel), which are all characteristic of the [Ni(salen)] complex.

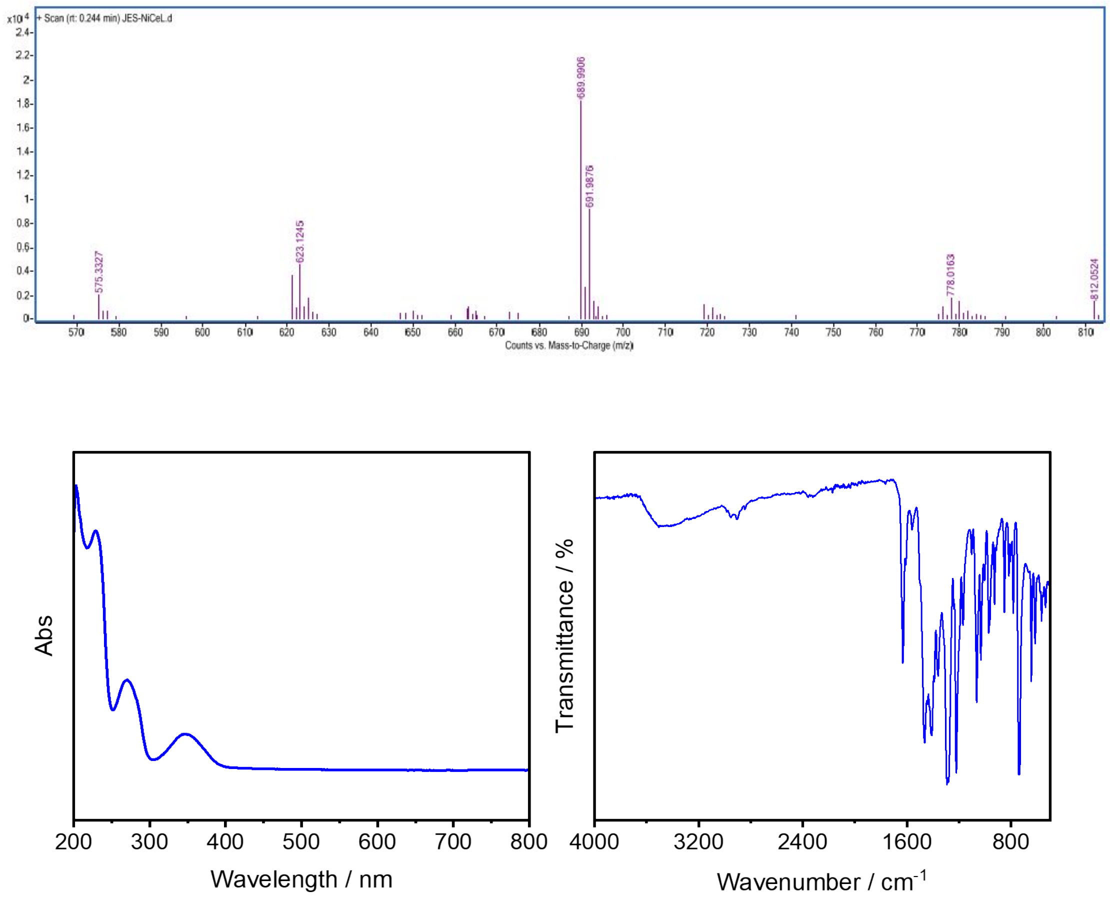

Figure S12. Cerium-Nickel complex **CeNiL.** HR-MS of **CeNiL**. m/z: calcd. for [M-NO_3^-] $C_{21}H_{28}CeN_4NiO_{12}$: 689.9900; found [M-$NO_3^-$] = 689.9906**.** UV-vis spectrum of **CeNiL** the spectrum shows a strong absorption band in the ultraviolet region. This absorption band may be due to the charge transfer between the 2p level of O_2^- and 4f levels of Ce^{4+}. The cerium oxide shows the strong absorption band around 360 nm (3.4 eV). FTIR spectrum: The IR spectroscopy characterizes the presence of OH group on the surface of the sample by its stretching frequencies at 3400 and 3509 cm^{-1}. The absorption peak at 2362 cm^{-1} may be due to atmospheric CO_2 adsorbed by the surface of the sample. The peaks observed at 674 cm^{-1} and 545 cm^{-1} are attributed to the antisymmetric Ce–O–Ce stretching vibration of the surface bridging oxide.

4. Electrochemical characterization

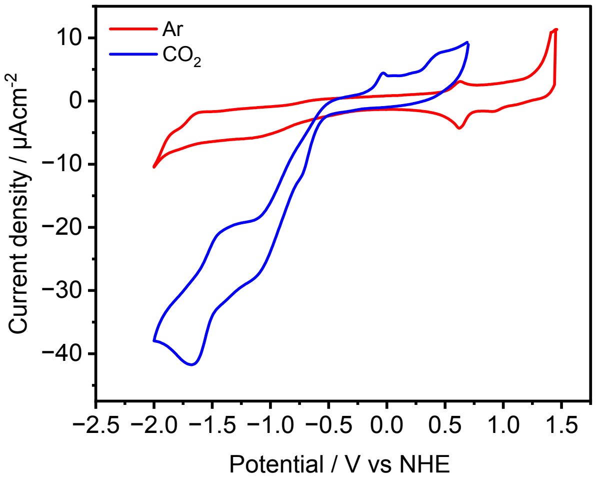

Figure S13. Comparison of cyclic voltammograms of **CeNiL** dissolved in acetonitrile under argon and CO_2 containing 0.1 M $TBAPF_6$ as supporting electrolyte with glassy carbon as working, platinum wire as counter and nonaqueous pseudo-Ag/AgCl as reference electrode with a scan rate of 30 mVs^{-1}

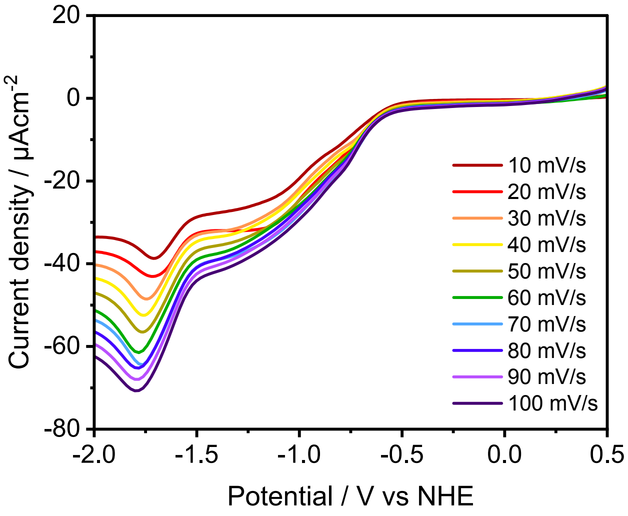

Figure S14. LSV curves of **CeNiL** dissolved in acetonitrile under argon and CO_2 containing 0.1 M $TBAPF_6$ as supporting electrolyte.

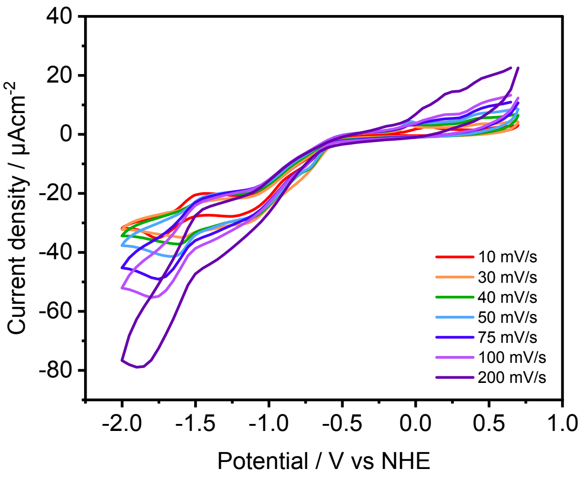

Figure S15. CV curves of **CeNiL** dissolved in acetonitrile under argon and CO_2 containing 0.1 M $TBAPF_6$ as supporting electrolyte.

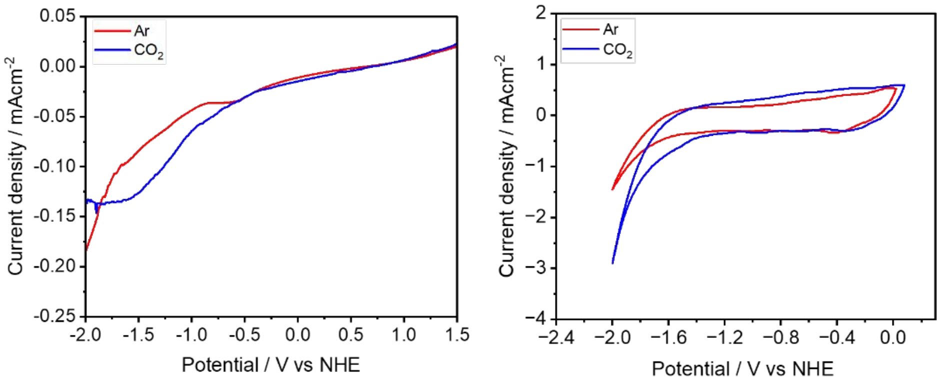

Figure S16. LSV and CV curves at a scan rate of 30 mV s^{-1} of **CeNiL**/CB catalyst modified carbon paper electrode with Ar and CO_2 saturation 0.1M $CsHCO_3$.

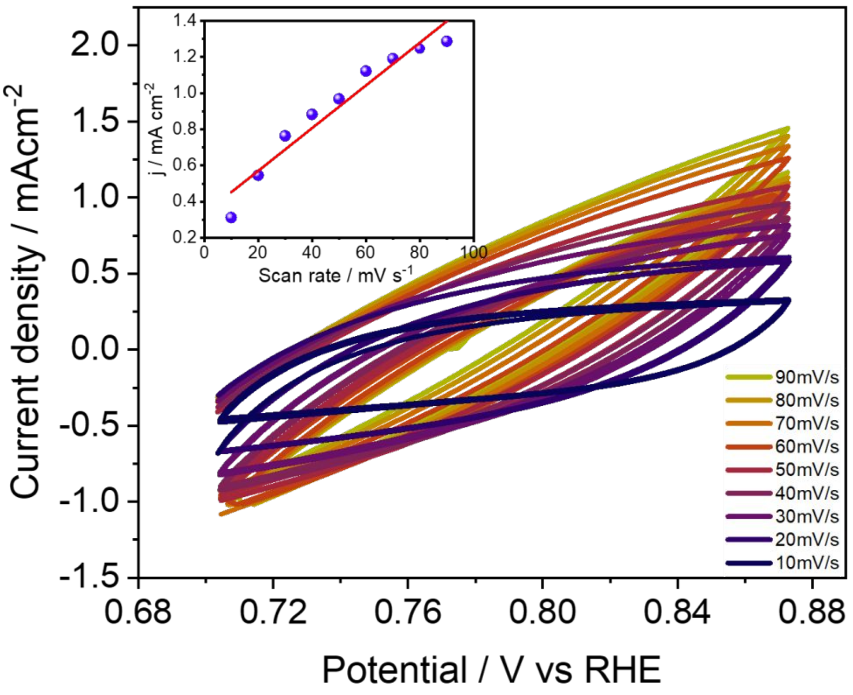

Figure S17. Cyclic voltammograms of **CeNiL**/CB catalyst modified carbon paper electrode at different sweep rates of 10, 20, 30, 40, 50, 60, 70, 80, and 90 mV s^{-1} from 0.70 to 0.87 V in 0.1M $CsHCO_3$ (b) a linear plot of capacitive current versus scan rate.

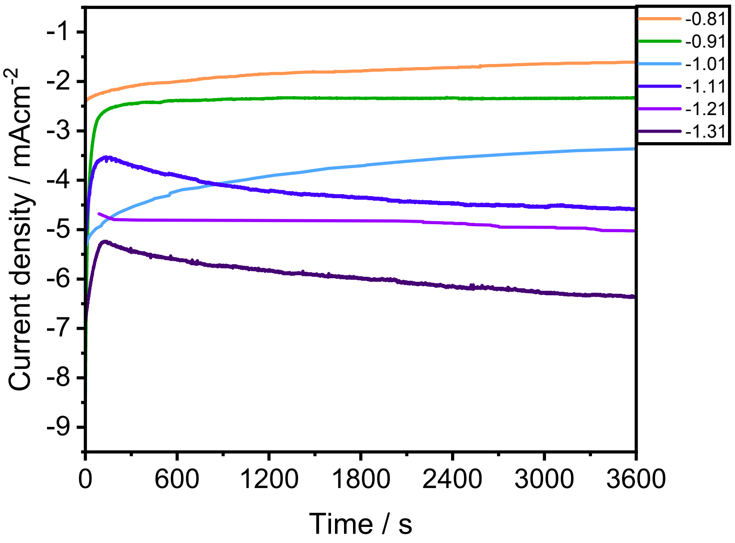

Figure S18. Cell current vs. time plot of **CeNiL**/CB catalyst modified carbon paper electrode at different half-cell potentials vs. RHE.

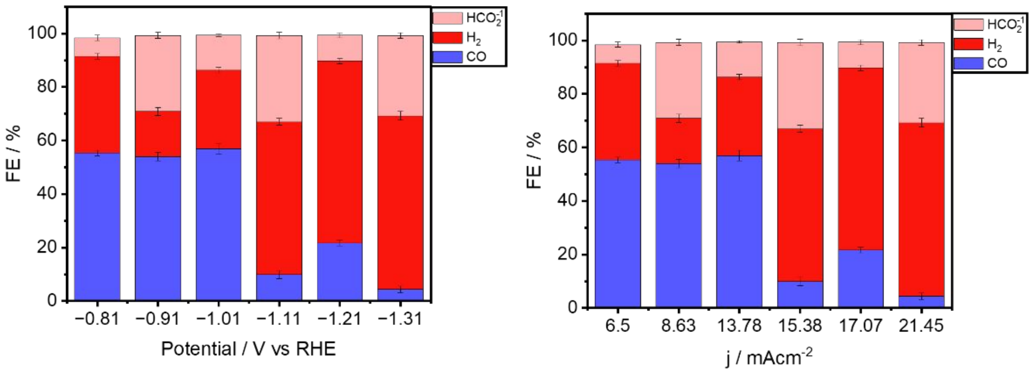

Figure S19. Performance of **CeNiL**/CB catalyst modified carbon paper electrode. a) Faradaic efficiencies for CO, H_2, and HCO_2^- obtained during one hour electrolysis at each potential displayed. b) Faradaic efficiencies for CO, H_2, and HCO_2^- obtained during one-hour electrolysis at each current density displayed

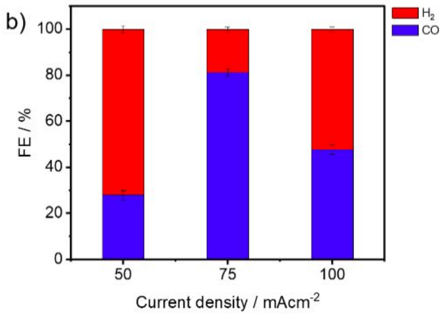

Figure S20. Comparison of the performance of **CeNiL**/CB catalyst for the CO_2 reduction in a zero-gap electrolyzer at 50, 75, and 100 mA cm^{-2} at 60°C after 24 hours of electrolysis. All investigated GDEs possessed a catalytic loading of 0.5 mg cm^{-2} of active material.

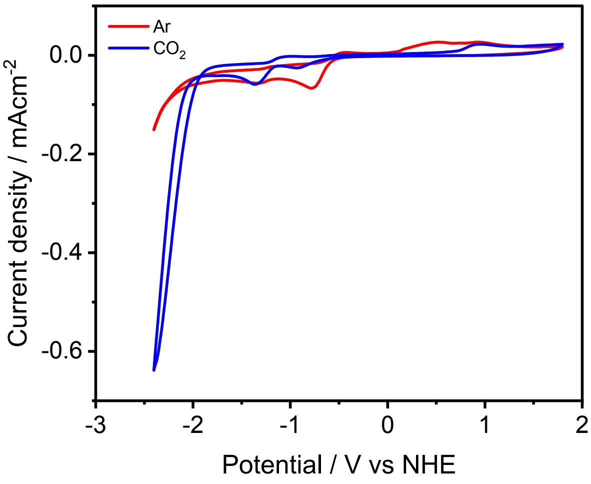

Figure S21. Comparison of cyclic voltammograms of **NiL** dissolved in acetonitrile under argon and CO_2 containing 0.1 M $TBAPF_6$ as supporting electrolyte with glassy carbon as working,

platinum wire as counter and nonaqueous pseudo-Ag/AgCl as reference electrode with a scan rate of 30 mVs^{-1}.

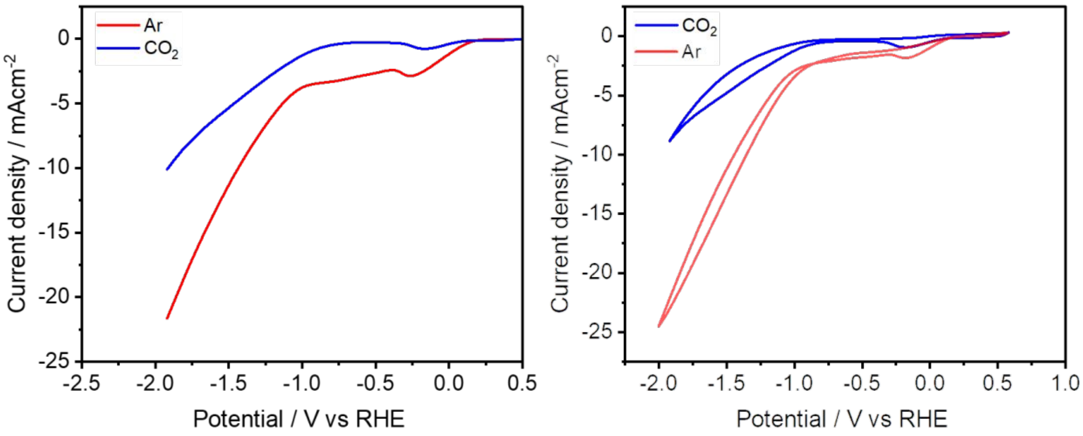

Figure S22. LSV and CV curves at a scan rate of 30 mV s^{-1} **NiL**/CB catalyst modified carbon paper electrode with Ar and CO_2 saturation.

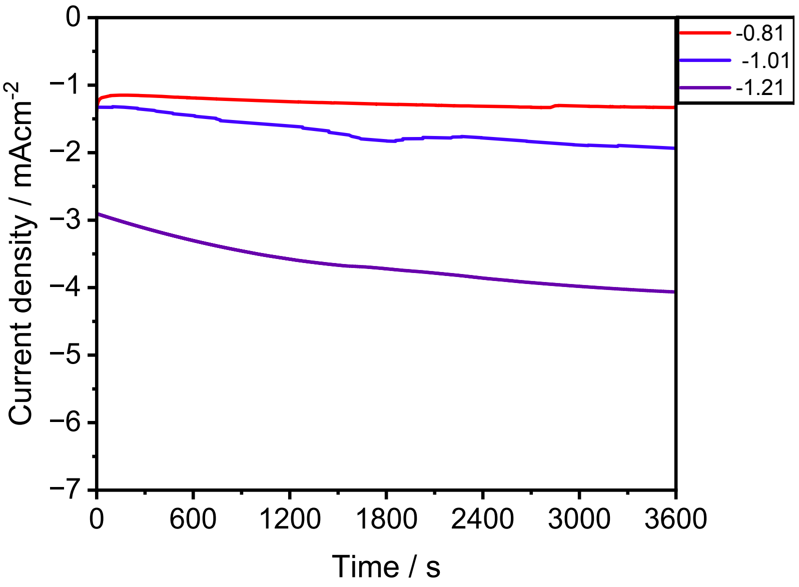

Figure S23. Cell current vs. time plot of **NiL**/CB catalyst modified carbon paper electrode at different half-cell potentials vs. RHE.

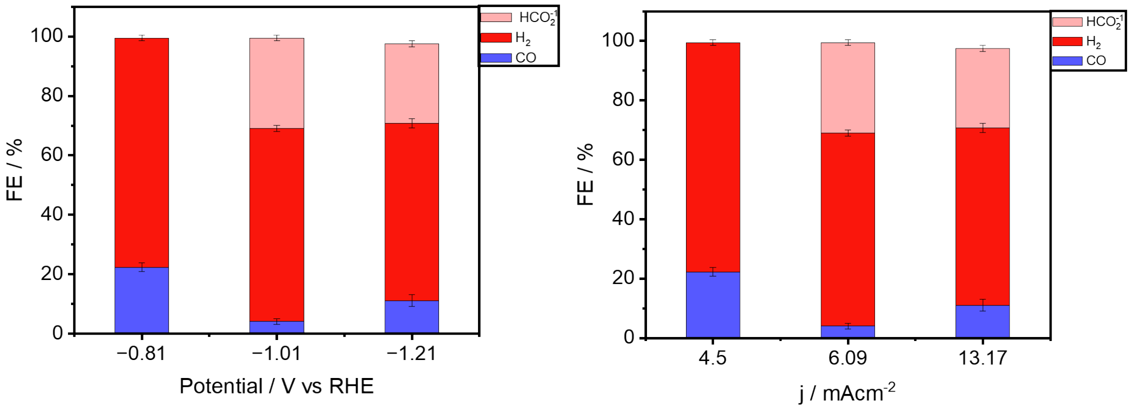

Figure S24. a) Faradaic efficiencies for CO, H_2, and HCO_2^- obtained during one-hour electrolysis **NiL**/CB catalyst modified carbon paper electrode at each potential displayed. b) Faradaic efficiencies for CO, H_2, and HCO_2^- obtained during one-hour electrolysis at each current density displayed.

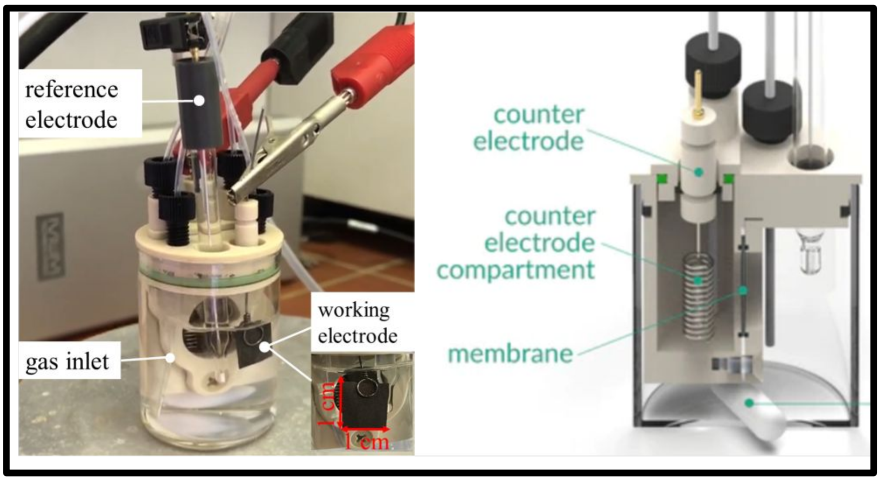

Figure S25. The electrolysis H-cell.

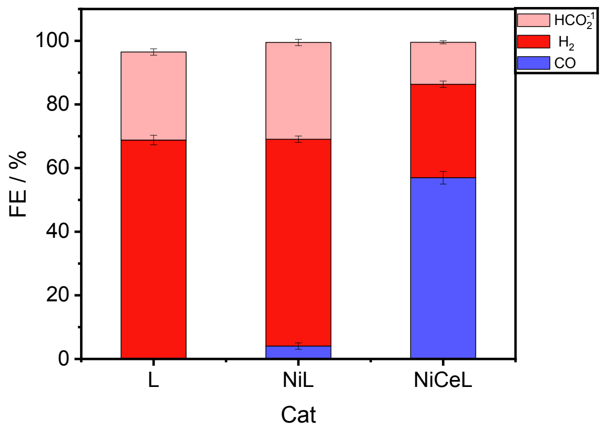

Figure S26. Performance of **CeNiL**/CB, **NiL/**CB, and **L**/CB catalyst WEs. Faradaic efficiencies of for CO, H_2, and HCO_2^- obtained during one-hour electrolysis at -1.0 V vs RHE.

5. Characterization by Electrochemical Impedance Spectroscopy (EIS)- Method

The electrochemical impedance spectroscopy (EIS) was measured using an IVIUM CompactStat (Netherlands). The impedance spectrum was recorded for all experiments in the frequency range of 10^5 Hz to 0.01 Hz with a perturbation amplitude of 10 mV. The aim of this characterization is the investigation of Ni(II)-Ce(III) diimine complex for the carbon dioxide reduction electrolysis, first two platinum electrodes were measured in a one-cell compartment with the corresponding electrolyte as a control experiment to determine the electrolyte resistance. Subsequently, the setup was transitioned to an H-cell configuration with a Nafion membrane, enabling the determination and subtraction of the membrane resistance from the electrolyte resistance. Further experiments involved replacing one platinum electrode with a carbon paper electrode as the working electrode. Lastly, the carbon paper, coated with **CeNiL**, served as the working electrode for the complete electrochemical cell evaluation through EIS. The resulting fitted and calculated impedance data, as well as resistance values for each cell component (electrolyte solution, membrane, carrier electrode) in the carbon dioxide reduction cell system are summarized in Table S1.

The Bode plot for the two-electrode system is also shown in the manuscript (Figure 1e). All resistance values for each cell component, i.e., electrolyte solution, membrane, and carrier electrode are shown in Table S1 for carbon dioxide reduction cell systems, respectively. Based on EIS, the applied electrochemical cells were characterized in detail indicating negligible losses of the systems.

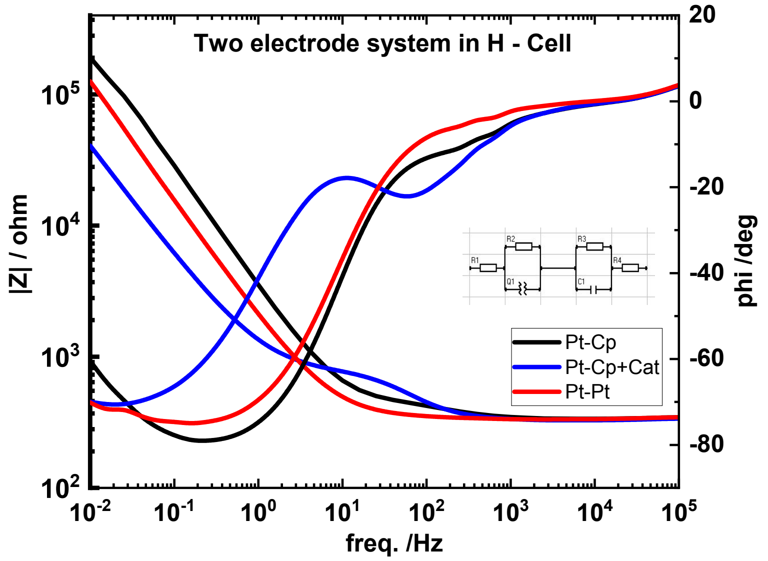

Figure S27. Bode plot recorded via electrochemical impedance spectroscopy of **CeNiL**/CB catalyst modified carbon paper electrode in the frequency range of 1*10^{-1} Hz to 1*10^{5} Hz with a perturbation amplitude of 10 mV.

Table S 1 Cell parameters extracted via electrochemical impedance measurements.

WE	CE	R_{Sol} / Ω	$R_{carrier}$ / Ω	R / $_{CeNi}$ Ω	R_{Me} / Ω	C $_{CeNi}$/ F	CPE-T	CPE-P
Pt	Pt	3.2E+01	6.5E+05	-	2.9E+02	-	9.5E-05	8.8E-01
GC	Pt	3.2E+01	5.7E+05	-	2.9E+02	-	5.4E-05	8.9E-01
CeNi	Pt	3.2E+01	1.5E+06	3.8E+02	2.9E+02	8.7E-06	2.4E-04	8.7E-01

6. Product analysis by GC-BID

Product analysis, conducted via Gas-Chromatograph Nexis GC-2030 by Shimadzu. During the electrolysis in the closed cell, 250 µL of the headspace gas was taken for the quantification of evolved gas. CO and H_2 were detected during the electrolysis in the cathodic region. For quantification purposes, the peak area was converted into a gas volume using the calibration curve.

The Faradaic efficiency (FE%) of the gas products can be quantified following this formula,

$$FE_i = \frac{z_i \cdot n_i \cdot F}{Q_{\text{total}}} = \frac{z_i \cdot \frac{f_{CO_2} \cdot t}{V_{\text{loop}}} \cdot x_i}{Q_{\text{total}}} \cdot F$$

where z_i, n_i, Q_{total}, f_{CO2}, t, V_{loop}, x_i, and F are the number of electrons involved in the reaction, the mole amount of the total product (mol), the total consumed charge (C), the flow rate of CO_2 (5 mL min^{-1}), the test time (60 min), the volume of the quantitative loop in GC instrument used for detection (0.25 mL), the mole amount of the product directly measured by GC instrument (mol) and the faradic constant (96485.33 C mol^{-1}), respectively.

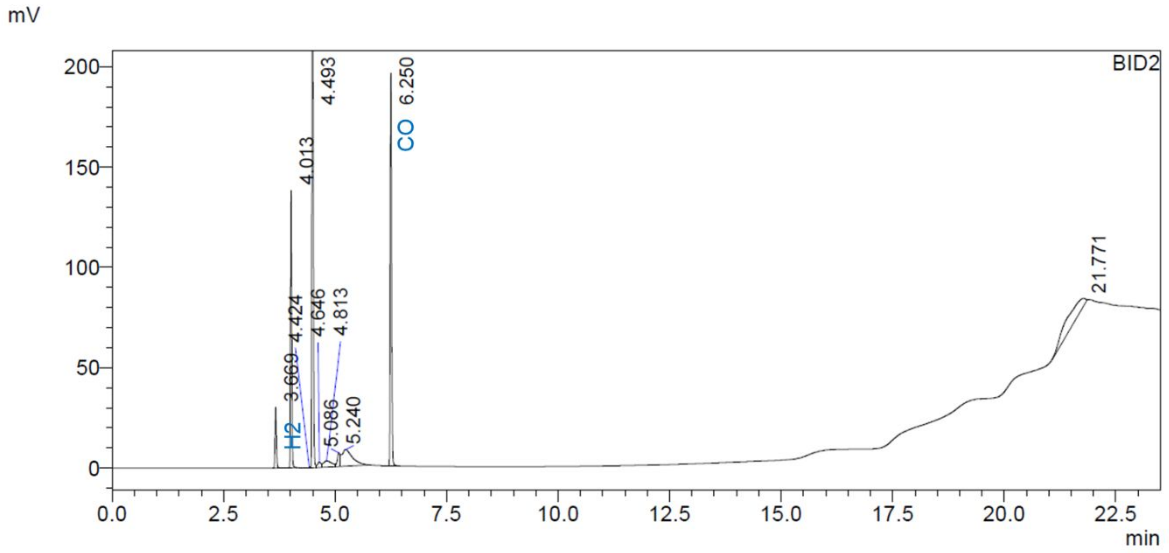

Figure S28. GC-BID-chromatogram gas products formed after CO_2 reduction at -1.01 V vs RHE for 1 h by **CeNiL**/CB catalyst modified carbon paper electrode in 0.1 M CO_2-saturated $CsHCO_3$ solution.

7. Product detection and quantification by ^{1}H-NMR

All liquid products have been quantified by a JEOL ECS-500 NMR spectrometer using D_2O as solvent. 50 µL of DMSO solution was used as internal standard. For this purpose, we have followed a previously reported procedure[10]. 450 µL analyte was taken directly from the reaction mixture for the ^{1}H-NMR analysis. Further suppression of the water peak was conducted in order to make the CO_2-reduced product peaks visible. Formate was found as only reduced product from ^{1}H-NMR with corresponding peaks at 8.2 ppm respectively (Figure S17). Number of scans and other spectral acquisition parameters were kept fixed during all acquisitions. During the quantification of the samples, each peak was normalized with respect to the DMSO peak at 2.7 ppm.

The Faradaic efficiency (*FE*, %) of the liquid products can be quantified according to this formula,

$$FE_i = \frac{z_i \cdot n_i \cdot F}{Q_{\text{total}}} = \frac{z_i \cdot \frac{V}{V_{\text{test}}} \cdot y_i}{Q_{\text{total}}} \cdot F$$

where y_i, V and V_{test} are the mole amount of liquid product directly measured by ^{1}H NMR spectra, the volume of total electrolyte (30 mL) and the volume of the tested electrolyte (0.2 mL), respectively.

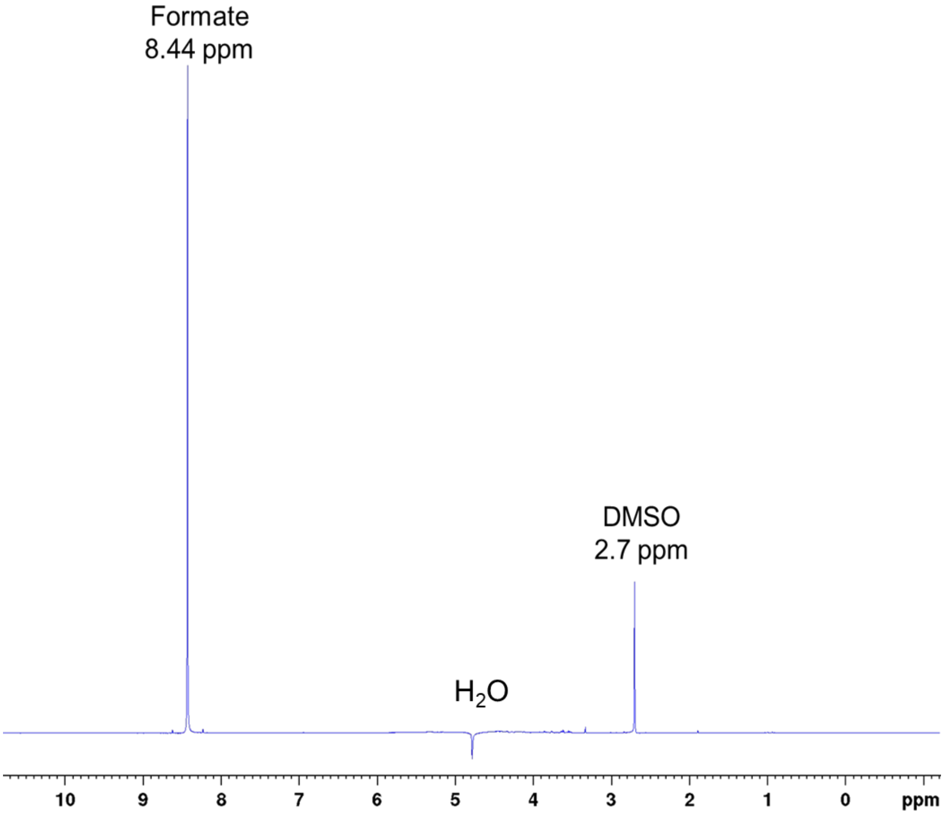

Figure S29. ^{1}H-NMR spectrum of the liquid products formed after CO_2 reduction at -1.21 V vs RHE for 24 h, by **CeNiL**/CB catalyst modified carbon paper electrode in 0.1 M $CsHCO_3$ CO_2-saturated solution.

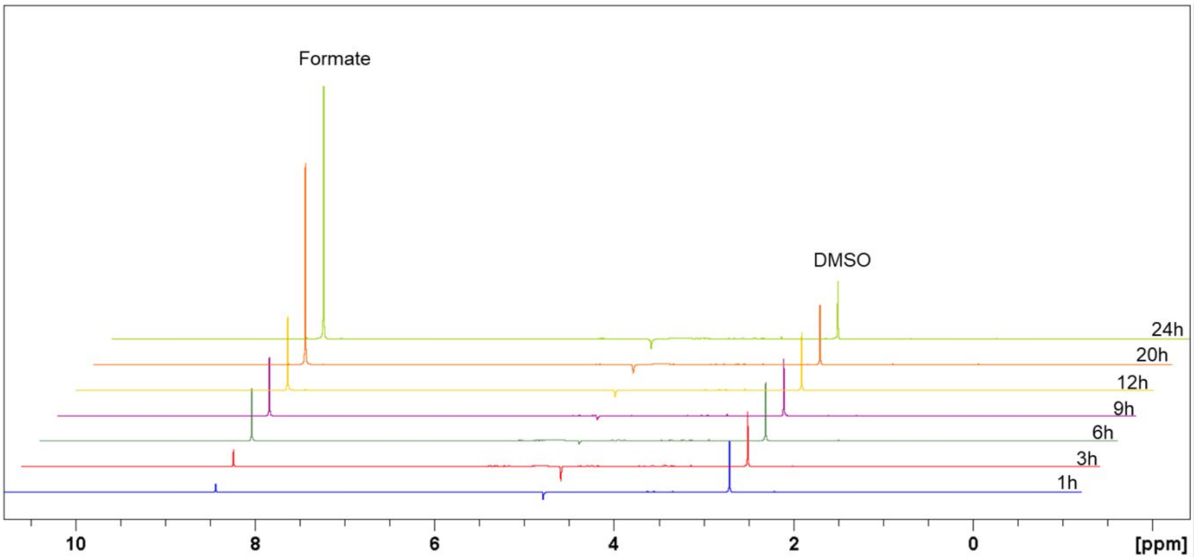

Figure S30. Time-dependent ^{1}H-NMR spectra of the liquid products formed after CO_2 reduction at -1.21 V vs RHE by **CeNiL**/CB catalyst-modified efficiency Carbon Paper electrode in 0.1M CO_2 -saturated $CsHCO_3$ solution.

TON TOF calculations

Turn Over Frequency Calculations:

Turn Over Frequency (TOF) was calculated using the equation[1];

$$\mathrm{TOF} = \frac{iE_F}{NFn_{cat}}$$

Where i = current

E_F = Faradaic efficiency for ethanol

N = Number of electrons in the half-reaction (N = 2 for CO_2 to CO conversion)

F = Faraday constant

n_{cat} = total moles of the catalyst employed for the electrolysis

Turn Over Number Calculations:

Turn Over Number was calculated using the equation;

$$\mathrm{TON} = \mathrm{TOF} \times t$$

8. Spectroelectrochemistry and chemical reduction experiments

the electrochemical redox behavior of CeNiL was studied under Ar and CO_2 atmosphere using spectroelectrochemical UV/vis (SEC-UV/vis) experiments. These experiments were conducted in an optical thin layer electrode cell (OTTLE cell), where a light transparent platinum mini-grid acted as working, as counter and a Ag-microwire as the pseudo-reference electrode. Experiments were pre-treated for 1 min at constant potential, with subsequent start of the UV/vis acquisition and electrolysis at identical conditions [3].

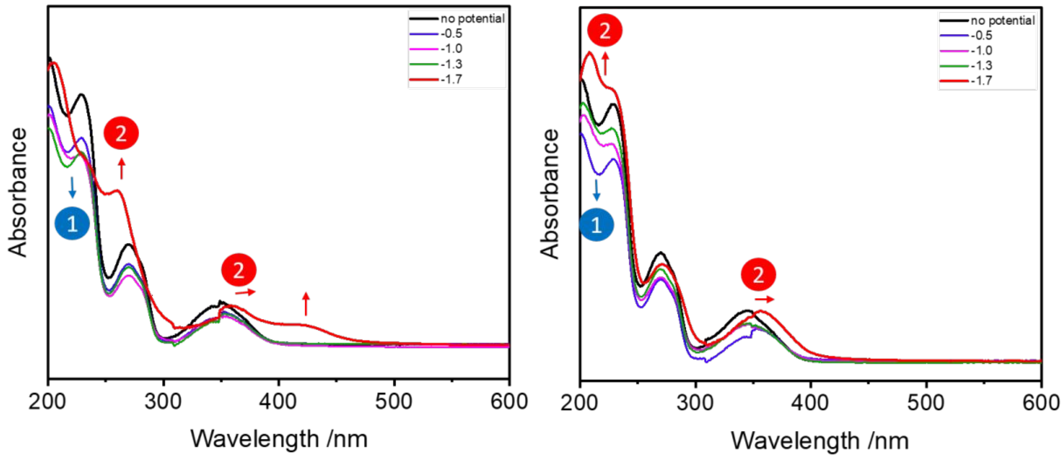

Figure S31. UV-vis spectroelectrochemistry of **CeNiL** in 0.2 M $TBAPF_6$ (in dry ACN), at potentials from -0.5 to -1.7 V vs Ag/AgCl. A) Ar, B) CO_2. Kinks at 350 nm stem from lamp changing of the UV-vis spectrometer.

For the chemical reduction tests an excess of KC_8 was added to **CeNiL** in an argon filled glovebox and then dissolved in dry acetonitrile for UV-vis spectroscopy. Afterwards a drop of water was added to this mixture via syringe. For the ^{1}H-NMR measurement aforementioned reactants were dissolved in non-dried CD_3CN.

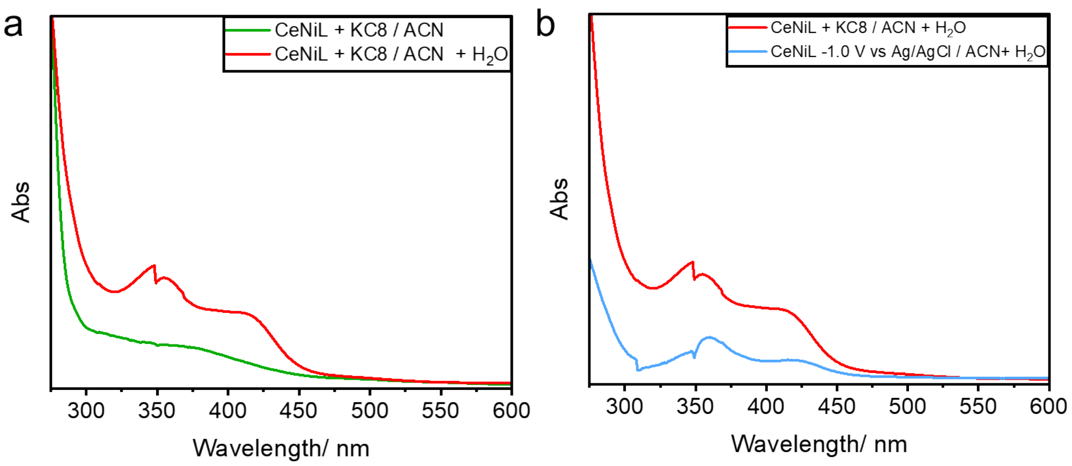

Figure S32. UV-vis of with KC_8 chemically reduced **CeNiL** under argon in ACN, ACN + a drop H_2O b) UV-vis of with KC_8 chemically reduced **CeNiL** under argon in ACN + a drop H_2O, 0.2M $TBAPF_6$ in ACN + H_2O under Ar at -1.0 V vs Ag/AgCl. Elevated absorption levels are caused by dispersed KC_8 particles.

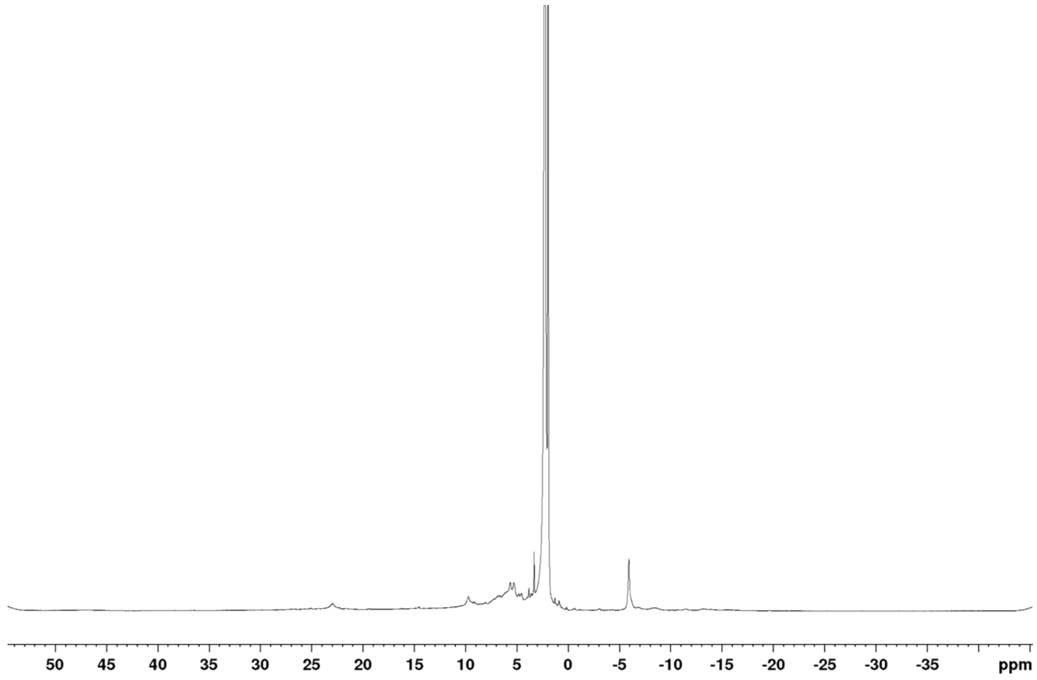

Figure S33. ^{1}H-NMR of with KC_8 chemically reduced **CeNiL** (500 MHz, CD_3CN, 25 °C).

9. Geometry Optimization, Frequency Calculations (Infrared and Raman)

CeNiL:

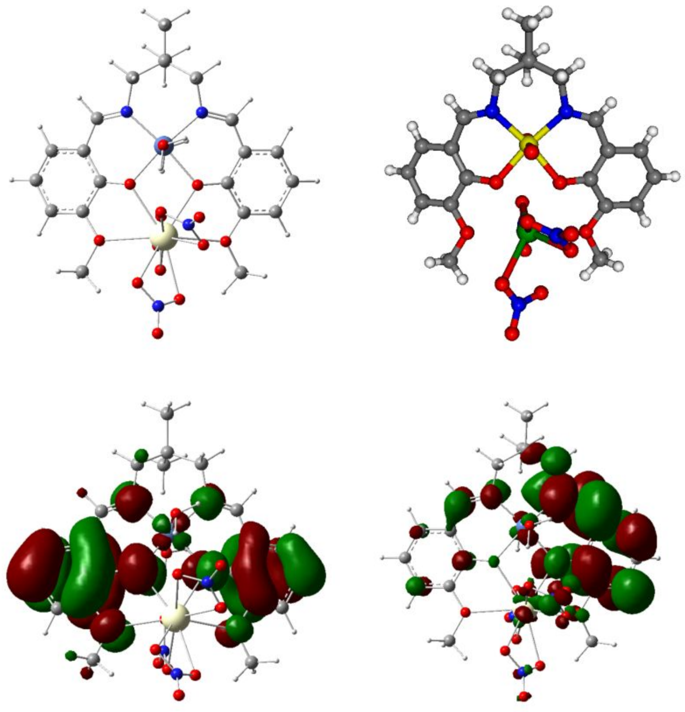

HOMO **LUMO**

Figure S34. Geometry optimized structure compared to molecular structure of dinuclear [**CeNiL**] as determined by single crystal X-ray diffraction analyses (axial coordinated water molecules are included). The axial ligands that are linked to the Ni center stem from the crystal water in the applied nickel precursor (cif file) and representations of frontier MOs of the **CeNiL** complex. (HOMO, including 15% Ni d_{yz}, d_{xy}).

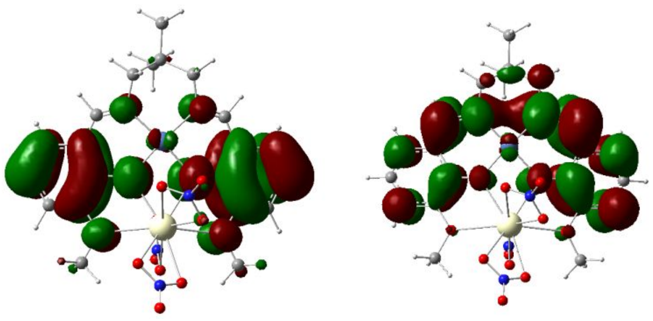

HOMO **LUMO**

Figure S35. Geometry optimized structure of dinuclear [**CeNiL**] without crystal water axially coordinated to nickel and representations of frontier MOs of **CeNiL** complex. (HOMO, including 15% Ni d_{yz}, d_{xy}).

NiL

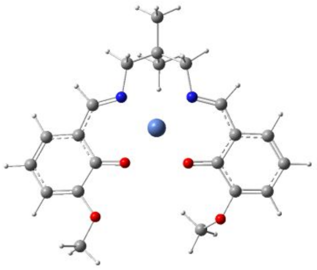

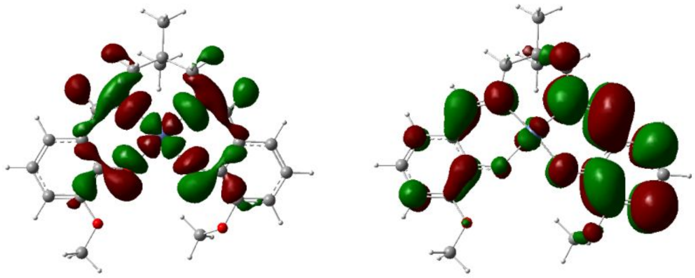

HOMO **LUMO**

NiL-$CO_2^{\cdot -}$

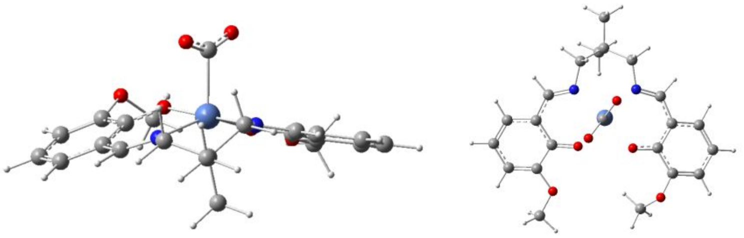

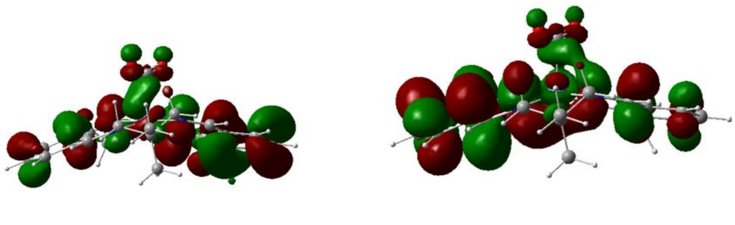

HOMO **LUMO**

Figure S36. Geometry-optimized structure and representations of frontier MOs of $Ni^{1+}L$-complex and geometry-optimized structure and representations of frontier MOs of NiL-$CO_2^{\cdot -}$ complex. (HOMO, 15% Ni dx^2-y^2, dxy).

CeNiL:

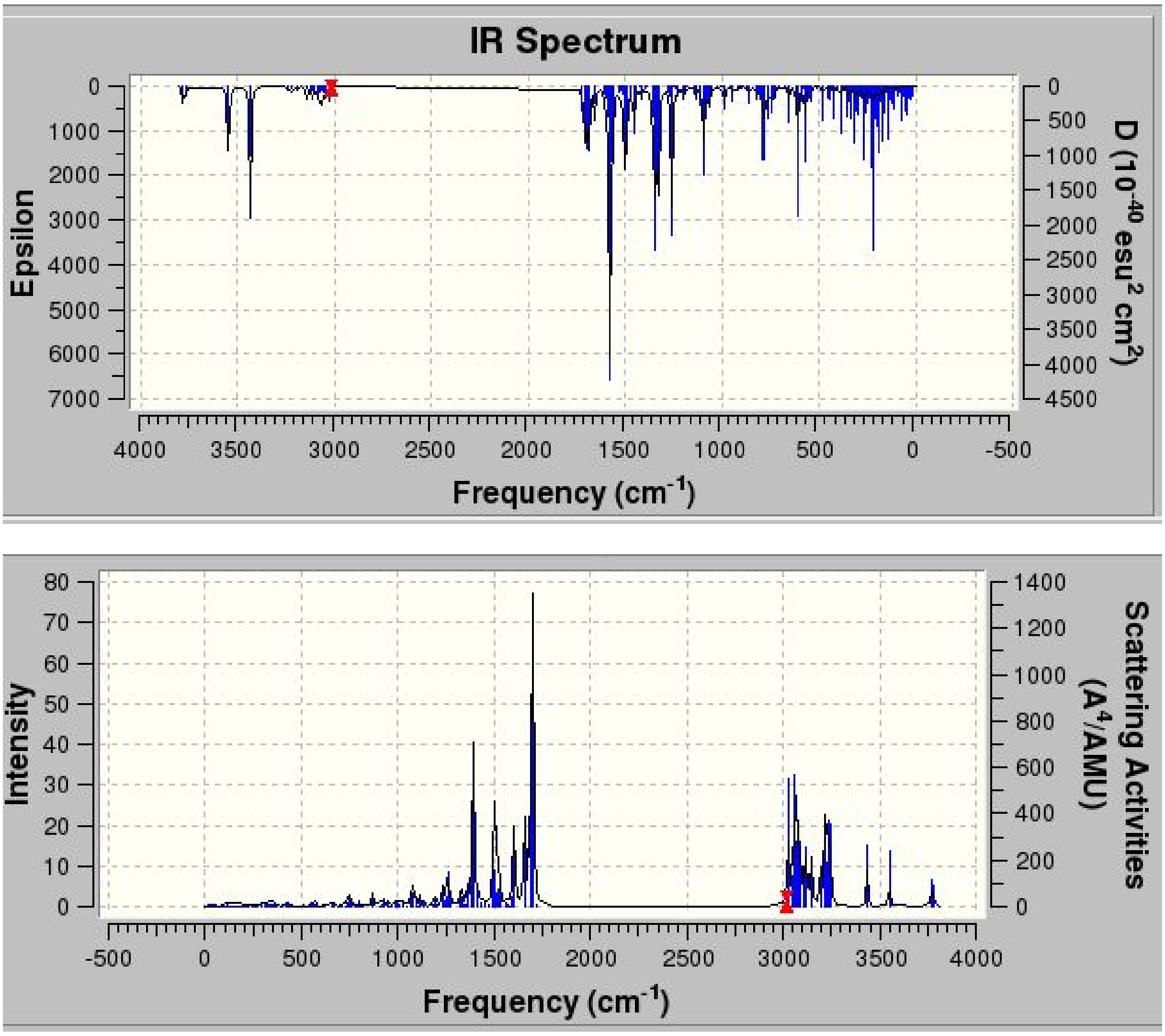

Figure S37. DFT calculated IR and Raman spectra of **CeNiL** complex (including axial coordinated water molecules).

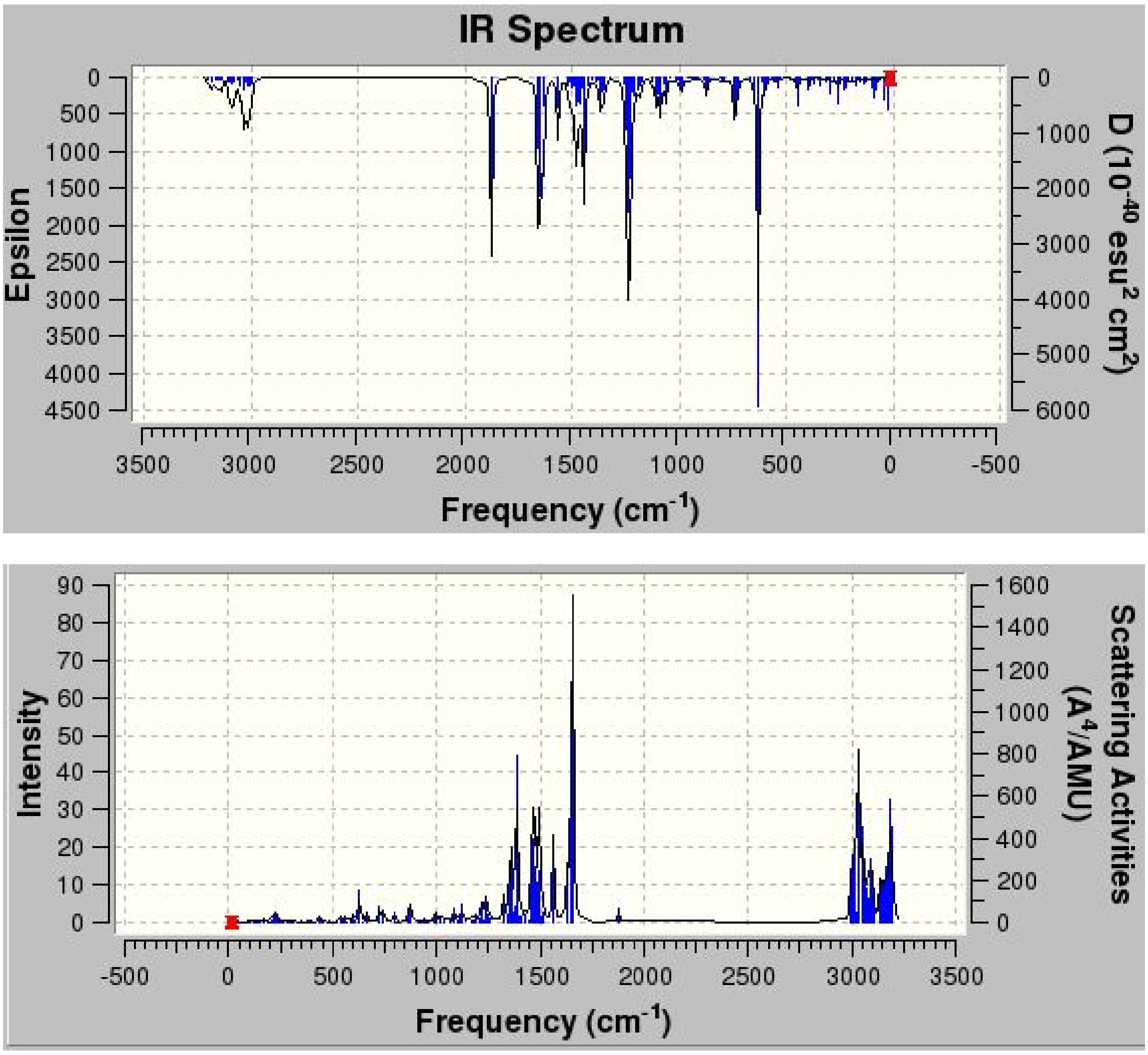

Figure S38. DFT calculated IR and Raman spectra of **CeNiL**-$CO_2^{\cdot -}$ complex.

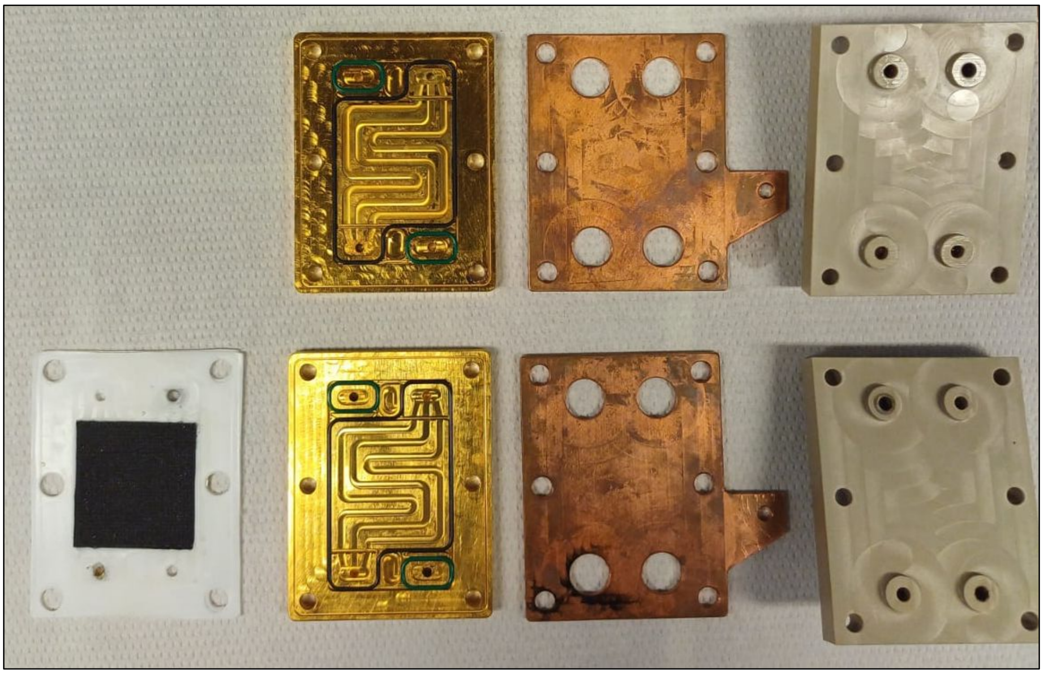

Figure S39. Zero-gap cell after electrocatalysis experiments**.**

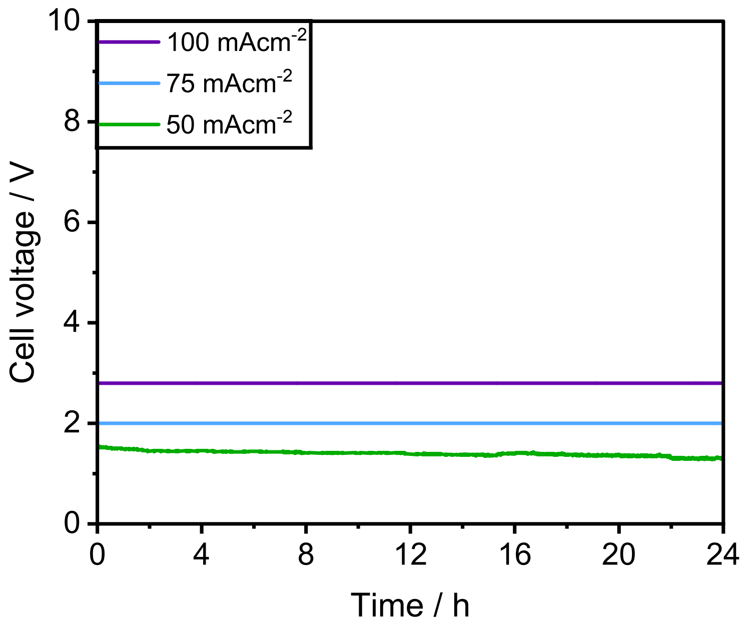

Figure S40. Cell voltage versus time plots for the conducted zero-gap cell electrocatalysis experiments**.**

10. References

[1] Michalke, J.; Faust, K.; Bögl, T.; Bartling, S.; Rockstroh, N.; Topf, C. Mild and Efficient Heterogeneous Hydrogenation of Nitroarenes Facilitated by a Pyrolytically Activated Dinuclear Ni(II)-Ce(III) Diimine Complex. *Int. J. Mol. Sci.* **2022**, *23*, 8742.

[2] Coskun, H.; Aljabour, A.; Luna, P. de; Farka, D.; Greunz, T.; Stifter, D.; Kus, M.; Zheng, X.; Liu, M.; Hassel, A. W.; Schöfberger, W.; Sargent, E. H.; Sariciftci, N. S.; Stadler, P. Biofunctionalized conductive polymers enable efficient CO2 electroreduction. *Sci. Adv.* **2017**, *3* (8), 68.

[3] Gonglach, S.; Paul, S.; Haas, M.; Pillwein, F.; Sreejith, S. S.; Barman, S.; De, R.; Müllegger, S.; Gerschel, P.; Apfel, U.-P.; Coskun, H.; Aljabour, A.; Stadler, P.; Schöfberger, W.; Roy, S. Molecular cobalt corrole complex for the heterogeneous electrocatalytic reduction of carbon dioxide. *Nat. Commun.* **2019**, *10* (1), 3864.

[4] Pellumbi, K.; Krisch, D.; Rettenmaier, C.; Awada, H.; Sun, H.; Song, L.; Sanden, S. A.; Hoof, L.; Messing, L.; Puring, K. j.; Siegmund, D.; Cuenya, B. R.; Schöfberger, W.; Apfel, U.-P. Pushing the Ag-loading of CO2 electrolyzers to the minimum via molecularly tuned environments. *Cell Rep. Phys. Sci.* **2023**, *4* (12), 101746.

[5] Bose, P.; Mukherjee, C.; Golder, A. K. A Ni II complex of the tetradentate salen ligand H 2 L NH2 comprising an anchoring –NH 2 group: synthesis, characterization and electrocatalytic CO 2 reduction to alcohols. *Inorg. Chem. Front.* **2019**, *6* (7), 1721–1728.

[6] Zheng, T.; Jiang, K.; Ta, N.; Hu, Y.; Zeng, J.; Liu, J.; Wang, H. Large-Scale and Highly Selective CO2 Electrocatalytic Reduction on Nickel Single-Atom Catalyst. *Joule* **2019**, *3* (1), 265–278.

[7] Bogart, J. A.; Lewis, A. J.; Medling, S. A.; Piro, N. A.; Carroll, P. J.; Booth, C. H.; Schelter, E. J. Homoleptic cerium(III) and cerium(IV) nitroxide complexes: significant stabilization of the 4+ oxidation state. *Inorg. Chem.* **2013**, *52* (19), 11600–11607.

[8] Jacobs, G.; Keogh, R.; Davis, B. Steam reforming of ethanol over Pt/ceria with co-fed hydrogen. *J. Catal.* **2007**, *245* (2), 326–337.

[9] Jia, Z.; Ning, S.; Tong, Y.; Chen, X.; Hu, H.; Liu, L.; Ye, J.; Wang, D. Selective Photothermal Reduction of CO 2 to CO over Ni-Nanoparticle/N-Doped CeO 2 Nanocomposite Catalysts. *ACS Appl. Nano Mater.* **2021**, *4* (10), 10485–10494.

[10] Grigioni, I.; Sagar, L. K.; Li, Y. C.; Lee, G.; Yan, Y.; Bertens, K.; Miao, R. K.; Wang, X.; Abed, J.; Won, D. H.; Garciá De Arquer, F. P.; Ip, A. H.; Sinton, D.; & Sargent, E. H. CO2 Electroreduction to Formate at a Partial Current Density of 930 mA cm-2with InP Colloidal Quantum Dot Derived Catalysts. *ACS Energy Let.* **2021**, *6* (1), 79-84.
